# Global fishing patterns amplify human exposures to methylmercury

**DOI:** 10.1073/pnas.2405898121

**Published:** 2024-09-23

**Authors:** Mi-Ling Li, Colin P. Thackray, Vicky W. Y. Lam, William W. L. Cheung, Elsie M. Sunderland

**Affiliations:** ^a^School of Marine Science and Policy, College of Earth, Ocean and Environment, University of Delaware, Newark, DE 19711; ^b^Harvard John A. Paulson School of Engineering & Applied Sciences, Harvard University, Cambridge, MA 02138; ^c^Institute for the Oceans and Fisheries, the University of British Columbia, Vancouver, BC V6T 1Z4, Canada; ^d^Department of Environmental Health, Harvard T.H. Chan School of Public Health, Boston, MA 02115

**Keywords:** fisheries, mercury, public health, seafood, tropics

## Abstract

Global pollution has exacerbated accumulation of toxicants like methylmercury (MeHg) in seafood. Human exposure to MeHg has been associated with long-term neurodevelopmental delays and impaired cardiovascular health, while many micronutrients in seafood are beneficial to health. The largest MeHg exposure source for many general populations originates from marine fish that are harvested from the global ocean and sold in the commercial seafood market. Here, we use high-resolution catch data for global fisheries and an empirically constrained spatial model for seafood MeHg to examine the spatial origins and magnitudes of MeHg extracted from the ocean. Results suggest that tropical and subtropical fisheries account for >70% of the MeHg extracted from the ocean because they are the major fishing grounds for large pelagic fishes and the natural biogeochemistry in this region facilitates seawater MeHg production. Compounding this issue, micronutrients (selenium and omega-3 fatty acids) are lowest in seafood harvested from warm, low-latitude regions and may be further depleted by future ocean warming. Our results imply that extensive harvests of large pelagic species by industrial fisheries, particularly in the tropics, drive global public health concerns related to MeHg exposure. We estimate that 84 to 99% of subsistence fishing entities globally likely exceed MeHg exposure thresholds based on typical rates of subsistence fish consumption. Results highlight the need for both stringent controls on global pollution and better accounting for human nutrition in fishing choices.

## Significance

Methylmercury (MeHg) is a potent neurotoxicant that adversely affects human health. Wild-caught marine species sold in the global commercial seafood market are the main MeHg exposure source for many populations. Here, we identify where and how much MeHg is extracted from the ocean during global marine fisheries harvests. We find that the geographic distribution of MeHg “fished” from the oceans predominantly reflects the harvesting locations of large pelagic species. Expansion of multinational industrial fisheries, particularly in low-latitude ecosystems, has exacerbated MeHg exposures for global seafood consumers. This work reveals that most subsistence fishing populations likely exceed exposure thresholds for MeHg and highlights the disproportionate impacts of global mercury pollution on subsistence fisheries in developing countries.

Marine seafood supplies essential protein and nutrition for billions of people globally and fishing offers income and employment for an estimated 10 to 12% of the world’s population (1). However, global pollution now means that seafood is the main mercury exposure source for many populations around the world (2, 3). Exposure to methylmercury (MeHg), the organic form of mercury that accumulates in food webs, is a well-established central nervous system toxicant (4). Even low levels of MeHg exposure have been associated with lasting developmental effects in children, especially those exposed during pregnancy (5, 6), and may increase cardiovascular health risks in adults (7, 8). Past research shows that the edible supply of seafood within a region provides a good approximation of population seafood intake patterns when compared to nationally representative dietary survey data (9) and can be used to reconstruct population-level MeHg exposures (2). Here, we extend this approach globally to analyze the sources of human MeHg exposure in the global commercial seafood market and for subsistence fishing entities.

Most of the seafood sold in the commercial market is harvested by industrial fisheries. Industrial fisheries are defined by their use of large, motorized vessels and sale of their catches predominantly in commercial markets. However, exposures to MeHg are known to be highest among subsistence fishers and indigenous populations (10). These populations mainly participate in small-scale fisheries that predominantly harvest fish in domestic waters (11) and consume locally caught seafood at much higher rates than other populations, thereby increasing their potential MeHg exposures.

MeHg concentrations in seafood vary by 2 to 3 orders of magnitude across and within marine species, depending on their trophic level (TL) and harvesting location (9, 12). Despite decades of research on mercury concentrations in fish, direct measurements are only available for a small fraction of the >1,700 species that are harvested and sold in the commercial seafood market (13, 14). Most available mercury measurements are either not resolved on a regional basis or represent site-specific contamination rather than central values likely to be observed in the global commercial seafood market (9, 13). This means that modeling approaches that leverage from available measurements are needed to fully reconstruct population-level MeHg exposures from fisheries.

Past research has proposed that micronutrients in seafood such as omega-3 fatty acids and selenium (Se) can offset potential MeHg exposure risks (15, 16). While this literature and supporting data are well established for omega-3 fatty acids (e.g., ref. 17), recent research has suggested that inadequate data are available for assessing the impacts of dietary Se on MeHg toxicity in humans (18). Nonetheless, comparing relative intakes of MeHg to micronutrients is important for simultaneously assessing nutritional risks and benefits of seafood (12).

The main objective of this work was to better understand the spatial origins and magnitudes of MeHg fished from the ocean for human consumption. We combined high-resolution data on spatially resolved fisheries catches (biomass) with empirically modeled seafood MeHg concentrations at the same locations to estimate the mass of MeHg extracted from the ocean in seafood for human consumption (hereafter referred to as “MeHg fished”). We compared the amounts of MeHg fished in exclusive economic zones (EEZs) around the world to the amounts of omega-3 fatty acids and Se in the same fisheries. This work improves our understanding of the seafood sources and spatial origins of MeHg and provides insights into relative intake of toxicants and nutrients in seafood harvested across marine regions. Such an analysis is essential for understanding how future changes in environmental quality are likely to affect marine foods.

## Results and Discussion

Our modeling approach for MeHg concentrations in marine species leveraged from a comprehensive effort to synthesize available data for the most important seafood categories in the commercial market (13) and the strong relationship between MeHg concentrations and TL. We used this analysis to derive the global grand mean and variability in MeHg concentrations of commercially important seafood in the commercial market (*Materials and Methods*). We spatially scaled measured values within their observed ranges using data on foraging territory of different seafood species (14) and modeled global MeHg concentrations in seawater (19). This means our global exposure estimates represent an empirically estimated central approximation and spatial variability in modeled estimates of MeHg concentrations are constrained to observed variability in seafood mercury concentrations.

We evaluated the reliability of our approach for spatial scaling of MeHg concentrations, which affect estimates of the spatial origin of MeHg. To do this, we used a large dataset (*n* = 1,482) for yellowfin tuna caught in 24 distinct marine regions (*SI Appendix*, Table S1). We chose this species because it has abundant geographical coverage, relatively constrained spatial migration, and large variability in observed mercury concentrations. Most of the >1,700 fish species harvested in the commercial seafood market are endemic, meaning they have limited geographic distribution and therefore lack the global distribution needed to evaluate our spatial modeling approach.

Our spatial modeling approach shows good predictive performance based on a weighted linear regression of observed and modeled yellowfin tuna mercury concentrations (R^2^ = 0.80; *SI Appendix*, Fig. S1). Most of the modeled values are within the empirical concentration range for common market sizes of yellowfin tuna (5 to 20 kg) (*SI Appendix*, Table S1). Lower predictive performance occurs in yellowfin caught in some coastal regions (*SI Appendix*, Fig. S1), likely reflecting spatial heterogeneity not captured in our global modeling approach due to data limitations for coastal ecosystems globally.

**Fig. 1.**
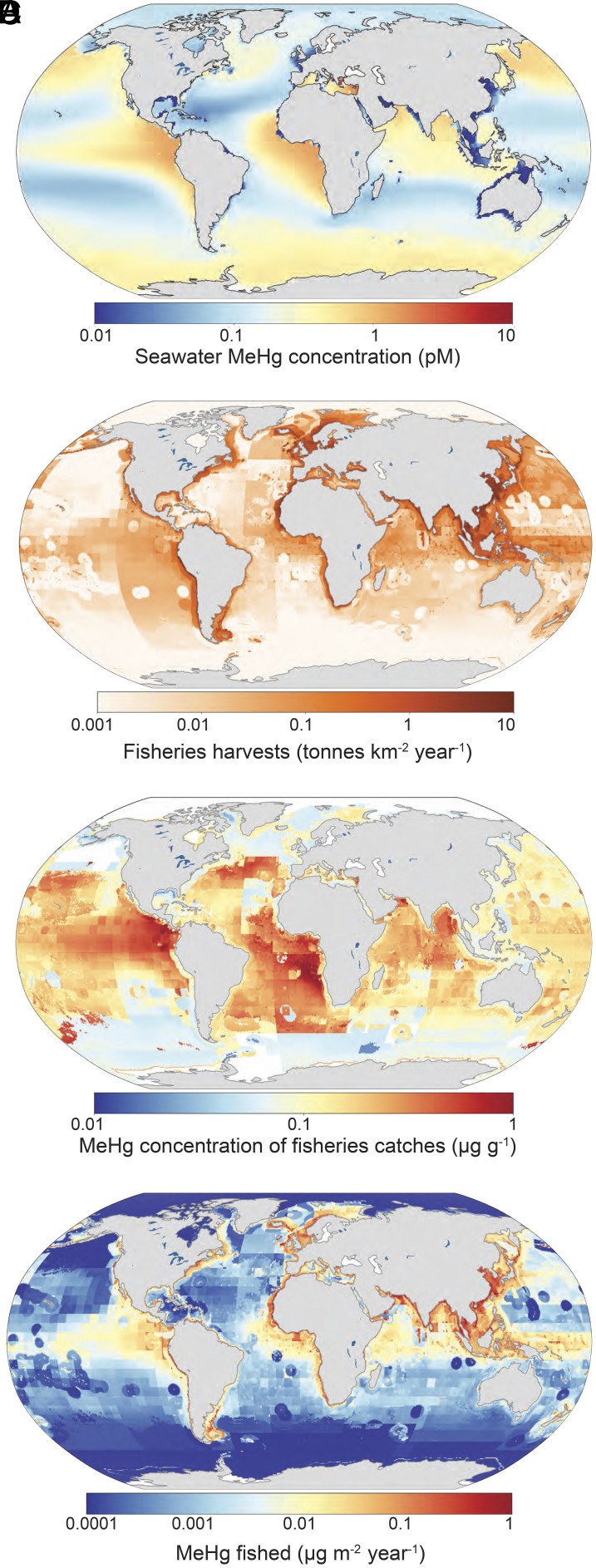
MeHg fished from the ocean. Panel *A* shows modeled seawater MeHg concentrations in the upper 1,000 m of the ocean from prior work (19). Panel *B* shows seafood harvests for direct human consumption between 2001 and 2010 at the 0.5° × 0.5° horizontal resolution for 1,774 species from the *Sea Around Us* database (14). Panel *C* shows modeled catch-weighted MeHg concentrations or each location. Panel *D* shows MeHg fished from the ocean annually through seafood harvests (blue = low and red = high). Spatially resolved seawater MeHg concentrations (*A*) and fisheries catches data (*B*) and their ecological traits were used to calculated catch-weighted seafood MeHg concentrations at each location (*C*) and the mass of MeHg fished (*D*) (*Materials and Methods*).

Across regions, our results suggest that catch-weighted MeHg concentrations in seafood vary by more than two orders of magnitude (range: 0.004 to 1.7 µg/g wet weight; geometric mean: 0.13 µg/g wet weight; Fig. 1*C*). Concentrations of MeHg fished from the ocean are lowest at high latitudes and greatest in the tropics and subtropics. Sensitivity analysis reveals this pattern occurs even when we prescribe a uniform spatial distribution of seawater MeHg (*SI Appendix*, Fig. S2 *A* and *B*). This implies the global distribution of MeHg fished is driven predominately by the combination of large harvesting biomass and biomagnification of MeHg in higher-trophic-level species.

The central estimate of MeHg extracted from the ocean by fishing accounts for 6.1 Mg (interquartile range [IQR]: 4.2 to 9.5 Mg; Fig. 1*D* and *SI Appendix*, Fig. S2 *C* and *D*). This estimate differs from past work that estimated MeHg extracted from the ocean in seafood and marine mammals at coarser spatial resolution and did not separate the edible supply of MeHg in seafood for human consumption (20). Past studies have estimated there is approximately 12,700 Mg of MeHg in the upper ~1,000 m of seawater (19). Our result suggests that fisheries harvests for human consumption account for ~0.05% of this reservoir (IQR: 0.03 to 0.08%) and do not appreciably deplete seawater MeHg concentrations.

### High MeHg Fished in Tropical and Subtropical Regions.

**Fig. 2.**
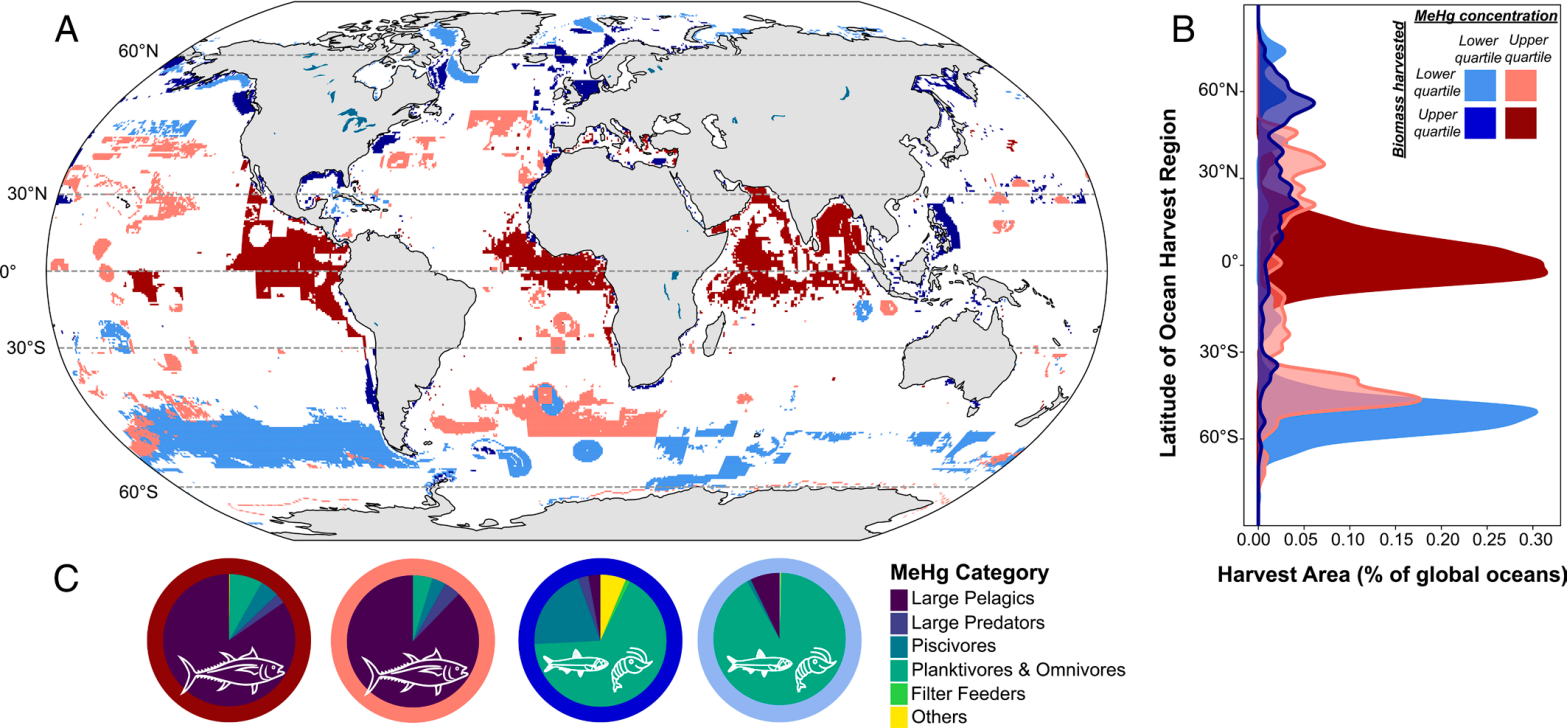
Overlapping upper and lower quartiles of seafood biomass harvests and catch-weighted MeHg concentrations. Panel *A* shows ocean regions with i) high MeHg and high catch (maroon), ii) high MeHg and low catch (pink), iii) high catch and low MeHg (dark blue), and iv) low catch and low MeHg (light blue). Panel *B* shows location of each combination as a function of ocean latitude. Panel *C* shows seafood types contributing to MeHg mass flux for each of the four high/low catch/MeHg quartiles.

We identified marine regions with overlapping upper and lower quartiles of seafood biomass harvests and catch-weighted MeHg concentrations (four categories highlighted in Fig. 2). Results show that the upper quartiles of catch biomass and fisheries MeHg are colocated in many low-latitude ocean regions, such as productive regions in the eastern equatorial Pacific and Atlantic oceans, tropical Arabian Sea, and the Bay of Bengal (Fig. 2, maroon shading). These regions produce large amounts of biomass for commercially important fisheries and are the main fishing grounds for large pelagic species like tunas. Large pelagic species account for approximately 60% of fisheries biomass and more than 80% of the total MeHg mass extracted from these ocean regions (*SI Appendix*, Table S2).

We compared our baseline results (Fig. 1) to a scenario with uniform seawater MeHg concentrations. Both results indicated that tropical and subtropical fisheries account for the largest fraction of MeHg fished from the ocean. The simulation scaled by seawater MeHg levels amplifies this effect due to relatively higher seawater MeHg concentrations in tropical and subtropical regions compared to other productive fishing grounds (*SI Appendix*, Fig. S3 *A* and *B*).

Prior work shows that the global oceans receive 70 to 80% of all atmospheric Hg deposition, and half of this is deposited between 30°N and 30°S (21, 22). This means that the tropics and subtropics have been disproportionately impacted by global anthropogenic Hg pollution. High primary productivity in many tropical and subtropical marine regions facilitates microbial activity in subsurface waters and enhances microbial methylation of inorganic Hg in seawater (19), thereby increasing the bioavailable Hg pool for food webs.

Large pelagic species such as tunas are top predators in low-latitude food webs and typically have an order of magnitude higher MeHg concentrations than lower-trophic-level fish (13). Pelagic predatory fish are most likely to exceed the regulatory limits for tissue MeHg concentrations established in the United States (23). Targeted fishing of large pelagic species and higher bioavailability of Hg at the base of the food webs results in the dominance of the tropics and subtropics as source regions for MeHg in global commercial seafood markets (24). Recent literature has noted that oligotrophic food webs tend to have more TLs and thus can exhibit the highest concentrations of MeHg in species from these regions (25). However, low productivity in the oligotrophic ocean and resulting low catch biomass means their contributions to the total MeHg fished from the ocean for the commercial seafood market is also low.

Coincident location of the lower quartiles of catch biomass and catch-weighted MeHg concentrations occur predominantly at higher latitudes in the Southern Hemisphere (Fig. 2, light blue shading). In the Northern Hemisphere oceans, high catches with lower MeHg are more common (Fig. 2, dark blue shading). Past research shows that seawater MeHg concentrations are sometimes elevated in high-latitude seawater due to enhanced stability of MeHg in colder waters, slowing demethylation (19) (Fig. 1*A*). However, productive commercial fisheries in mid-latitude and subarctic regions are dominated by Alaska pollock and small forage fishes (e.g., herring), which are approximately an order of magnitude lower in MeHg than the large pelagic fish caught in the tropics (13). Many coastal regions globally have high fisheries yields but mainly supply shellfish and low mercury fish such as anchovies to the commercial market (*SI Appendix*, Table S2).

Our analysis of catch data suggests that industrial fishing accounts for an estimated 76% of the edible seafood biomass fished from the ocean. The current geographic distribution of MeHg fished largely reflects the spatial pattern of large pelagic fisheries (tuna and tuna-like species, billfish like swordfish and marlin, and sharks) at lower latitudes (Fig. 1*D*). We find that large pelagic species are the greatest contributor to MeHg fished for human consumption (36%; IQR: 36 to 38% of total MeHg) among seafood categories.

Technology and market-driven trends in industrial fisheries explain the current dominance of large pelagic fisheries as the predominant MeHg source in marine capture fisheries. Total global seafood biomass harvested by wild-caught marine fisheries has declined since 1996 (11). By contrast, the presence of large pelagic seafood in commercial markets has increased in recent decades and has been driven by advances in fishing techniques, such as onboard freezing capacity and fish aggregating technology (26). These technologies have substantially expanded offshore industrial fishing of large pelagic species (26). For example, global tuna catches grew between 1950 and 2014, followed by a plateau in recent years (27–29) and many countries have developed important tuna fisheries since the 1980 s due to the accessibility of advanced fishing technologies (28). Increased supply of these fish has greatly increased market availability and human consumption of seafood products like canned and fresh tuna (26).

### Higher Mercury-to-Micronutrient Ratios in Low Latitude Regions.

**Fig. 3.**
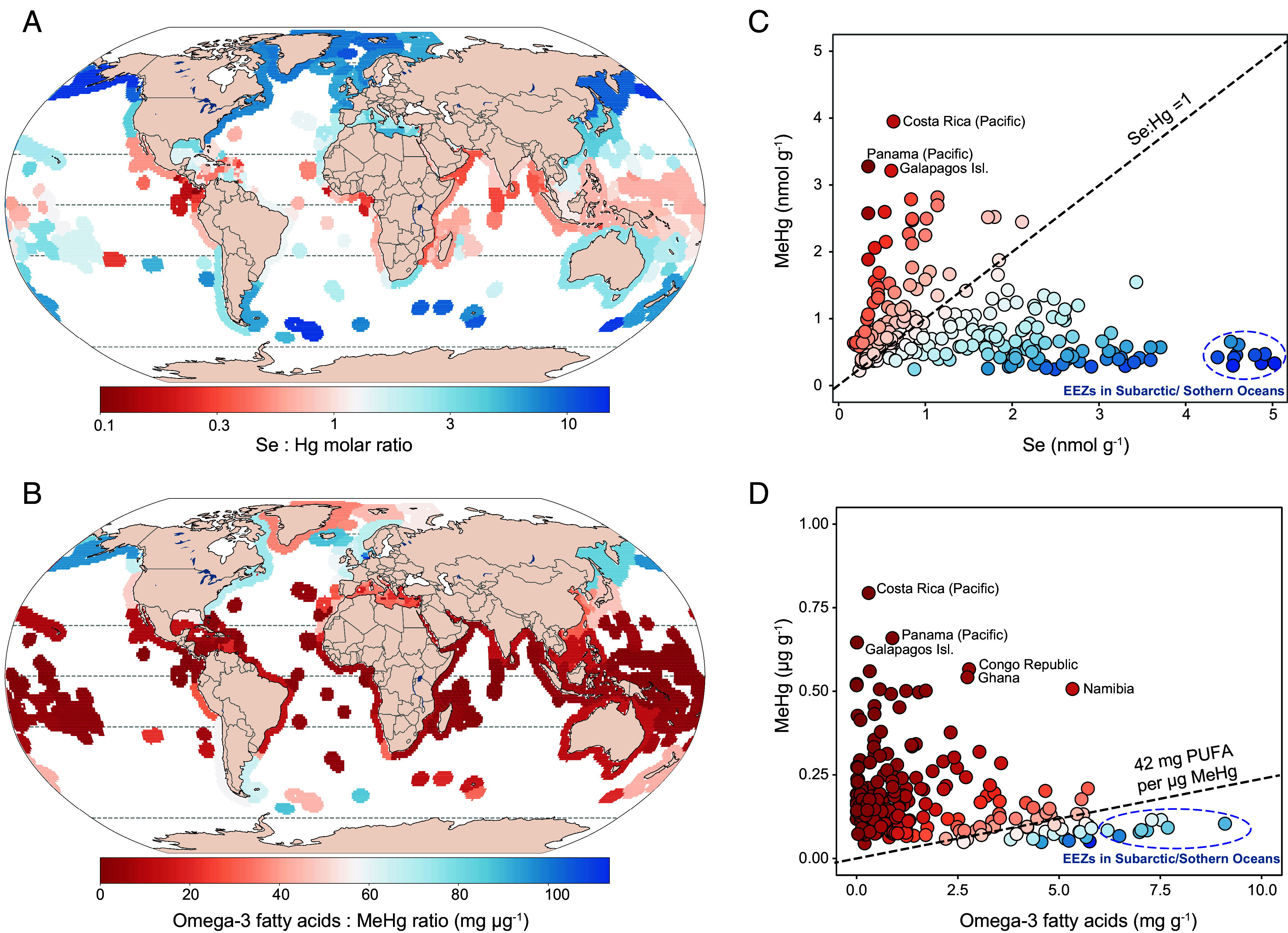
Ratios of micronutrients to MeHg in fisheries catches from different global regions. Data are shown for 242 EEZs globally for catches between 2000 and 2010. Panels *A* and *B* show the average ratios of micronutrients (omega-3 fatty acids and selenium: Se)-to-MeHg for the catch-weighted fisheries harvest in each EEZ. Panels *C* and *D* show ratios for fishery catches from individual EEZs (circles). Circle colors correspond to panels (*A* and *B*). The dashed line in panel *C* denotes the 1:1 Se:Hg molar ratios that may confer protection against the potentially deleterious effects of MeHg exposure. The dashed line in panel *D* shows the *de minimus* intake ratio for a 60 kg individual (42 mg omega-3 fatty acids per 1 µg MeHg) that allows them to meet their recommended nutritional requirements (250 mg of EPA and DHA per day) but maintain MeHg exposures below the US EPA RfD (0.1 µg/kg per day). *SI Appendix*, Table S2 provides raw data for concentrations of MeHg, Se, and omega-3 fatty acids in catches from each EEZ.

We calculated spatially averaged MeHg concentrations in seafood harvests from 242 EEZs and compared them to corresponding omega-3 fatty acids and Se concentrations in fisheries catches that were previously published (30) (*Materials and Methods*, Fig. 3 and *SI Appendix*, Table S3). Spatial averaging across coarse grid cells in global models can lead to an underestimate of seawater MeHg concentrations in coastal waters. To minimize this potential bias, we scaled modeled MeHg concentrations by empirical values for MeHg in coastal waters, where available (*Materials and Methods*). Sensitivity analysis of model results demonstrates that estimated ratios of MeHg to micronutrients in fisheries catches of each EEZ remain robust to different scaling assumptions (*SI Appendix*, Fig. S4 and section 1).

Past work has suggested that Se may confer protection against MeHg toxicity in humans and wildlife by facilitating MeHg demethylation and mercury biomineralization (15). However, the strength of evidence of this conclusion has been questioned in recent work (18). Regardless of its interactions with mercury, Se is an essential micronutrient at lower levels and confers benefits for human health, including optimal thyroid function and antioxidant properties (31). Se concentrations are substantially enriched at the base of food web but do not consistently biomagnify through higher trophic level fish (32).

For the purposes of visualizing the relative abundance of MeHg and Se in global fisheries, Fig. 3 shows the areas where seafood harvests exceed a molar ratio of Se:Hg >1 in blue and those where the molar ratio is <1 in red. Highest Se:Hg ratios are found in regions where low-trophic-level species are harvested and lowest ratios are found in regions that emphasize large pelagic catches (Fig. 3). More than half (57%; IQR: 46 to 71%) of global EEZs exhibit Se:Hg molar ratios >1, compared to only 30% (IQR: 24 to 59%) in tropical EEZs (Fig. 3 *A* and *C*). Lowest Se:Hg molar ratios are found in the EEZs of tropical and subtropical countries like Costa Rica, Panama, and the Galapagos Islands. By contrast, EEZs in the Subarctic and near the Southern Ocean tend to have the highest Se:Hg ratios (Fig. 3*C*).

Past work has highlighted the essential roles of eicosapentaenoic acid (EPA) and docosapentaenoic acid (DHA) (omega-3 fatty acids) for human brain and retinal tissue development and cardiovascular health protection for adults (12). EPA and DHA cannot be synthesized by the human body and therefore must be supplied by a dietary source. Marine phytoplankton synthesize these long-chain omega-3 fatty acids during carbon fixation, providing a dietary source for fish and shellfish, which are in turn, are the most common sources of EPA and DHA in the human diet (12). Concentrations of EPA and DHA vary across seafood species but are not correlated with MeHg concentrations (33). For example, large pelagic fishes like tuna, swordfish, and shark often have the highest MeHg concentrations among commonly consumed seafood but only provide low to moderate amounts of omega-3 fatty acids (12).

The Fig. 3*D* shows the *de minimus* ratio for omega-3 fatty acids and MeHg (16) that represents the amount of seafood an individual would need to consume to meet their nutritional requirements (250 mg of EPA plus DHA per day) but maintain their MeHg intake below the United States Environmental Protection Agency (U.S. EPA) Reference Dose (RfD) of 0.1 μg per kg body weight (BW) per day (34). This simple ratio was developed by state-level public health practitioners to communicate with the public about the risks and benefits of different seafood choices (16). Higher intake of EPA and DHA (300 mg/d) is recommended for pregnant and lactating women compared to the general population and for individuals with cardiovascular disease (up to 2,000 mg/d) (34). The RfD is defined as a lifetime MeHg exposure level that confers no appreciable increase in risk. We used the U.S. EPA RfD in this study because it applied a more conservative uncertainty factor of 10 to protect sensitive groups compared to other international groups (12).

We find that subpolar and polar EEZs are the only coastal regions globally where catch-weighted concentrations of omega-3 fatty acids and MeHg exceed the *de minimus* ratio (blue regions in Fig. 3 *B* and *D*). This means that most global fisheries within EEZs provide insufficient omega-3 fatty acids to avoid a net risk from MeHg intake. We infer from these results that global pollution of contemporary fisheries has made it difficult to meet nutritional requirements for EPA and DHA, while maintaining MeHg exposures below the U.S. EPA RfD.

Past experimental studies have shown that lower amounts of unsaturated fatty acids are required to maintain membrane fluidity in warmer water temperatures (35). Field observations confirm that plankton living in cold regions (−2 °C) contain up to three-fold greater unsaturated fatty acid concentrations compared to those in warm waters (29 °C) (35). The highest concentrations of omega-3 fatty acids occur in fish harvested from high latitudes, while the lowest concentrations are found in the tropical oceans (30). Prior work suggests that future ocean warming may further deplete production of omega-3 fatty acids by algae (35). Our results show that current concentrations of omega-3 fatty acids in most global fisheries are already insufficient for offsetting the deleterious effects of MeHg exposure. This problem may be exacerbated by future anthropogenic Hg pollution (36) and ocean warming (36, 37).

Our work reveals that current global fishing patterns amplify public health concerns associated with MeHg exposures. Current fishing practices extensively harvest species from low-latitude regions that have the highest MeHg concentrations due to a combination of global pollution, efficient biomagnification in high-trophic-level species, and natural biogeochemistry facilitating production of bioavailable Hg (Figs. 1 and 2). These same low-latitude fisheries offer lower nutritional benefits from Se and omega-3 fatty acids relative to MeHg levels compared to other regions globally (Fig. 3). Net nutritional benefits relative to MeHg exposures from Se and omega-3 fatty are only apparent in certain subpolar and polar coastal areas (Fig. 3). Human populations that rely on locally sourced seafood from lower latitudes, such as small island nations, may be particularly vulnerable to health risk associated with MeHg exposure.

Seafood is one of the most traded commodities globally, and accurate import and export data are needed to further characterize the population health impacts of MeHg exposure from marine fisheries. From solely a nutritional perspective, it would be beneficial to shift current fishing practices toward small pelagic fishes like anchovies, sardines, and herring that are higher in omega-3 fatty acids and lower in MeHg content (12). These species are already heavily harvested by industrial fishing fleets to produce animal feed and fish oil (38) and redirecting their use for direct human consumption could offer more nutritious options. However, we acknowledge that the benefits of fisheries on human well-being through economic livelihood and cultural preferences are not well-addressed by such simple substitutions. Minimizing global pollution remains the best long-term strategy for safeguarding the quality of wild-caught seafood.

### Subsistence Fisheries and Inequity in Mercury Exposures.

Subsistence fisheries are defined as those that produce locally harvested seafood to feed the families of fishers and the local community (11). The average seafood consumption rates reported for subsistence populations and coastal indigenous communities are much higher than those for the general population of various countries [>170 g seafood per person per day (39–41) compared to <20 g seafood per person per day (2)].

**Fig. 4.**
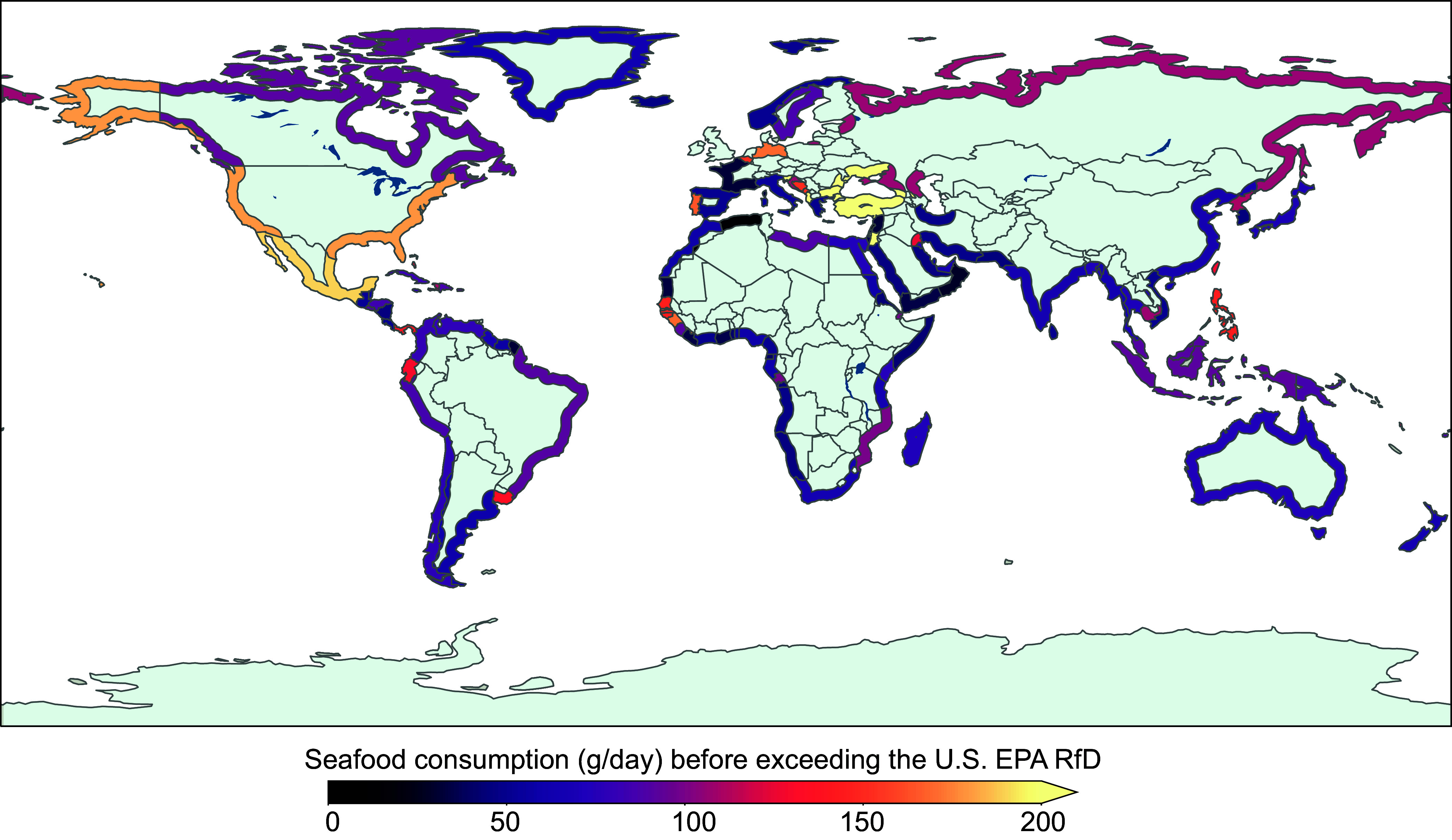
Estimated subsistence fish consumption without appreciable risk of MeHg exposure. Colored coastal regions indicate the amount of seafood (g/d) that can be consumed by individuals in each of the 173 marine subsistence fishing populations before exceeding the U.S. EPA RfD (0.1 µg/kg per day), based on their catch-weighted MeHg concentrations (*Materials and Methods*). *SI Appendix*, Table S4 provides mean MeHg concentration in catches for each marine subsistence fishing population and rates of seafood consumption that can be maintained without exceeding the U.S. EPA RfD.

Results of our analysis suggest that subsistence fish consumption rates in many countries are likely to result in exceedances of the U.S. EPA RfD for MeHg exposure due to current global pollution levels (Fig. 4 and *SI Appendix*, Fig. S5). MeHg concentrations in catch-weighted subsistence fisheries harvests range from 0.01 to 0.48 μg/g wet weight across 173 subsistence fishing entities. We estimate that 97% of these subsistence fishing entities (IQR: 84 to 99%) will exceed the U.S. EPA RfD for MeHg at 170 g of daily seafood intake by individuals (Fig. 4 and *SI Appendix*, Table S4 shows statistics for all countries). Variability across populations is driven by differences in species harvests across fishing regions and their corresponding MeHg burden (*Materials and Methods* and *SI Appendix*, Table S5). Our analysis is consistent with past work showing that subsistence fishers have among the highest blood Hg concentrations globally due to frequent fish consumption (10). This work provides screening-level insights into catch-weighted MeHg for subsistence populations in different global regions. However, more precise characterization of population exposures and risks in vulnerable regions based on direct measurements rather than modeling is needed. Such work should be based on human biomonitoring data, self-reported dietary preferences, and direct measurements of mercury concentrations in commonly consumed seafood species.

Past work shows that cumulatively since 1500, the vast majority of anthropogenic Hg emissions have originated in North America, Europe, and Asia in regions that are far from tropical and subtropical subsistence fishing populations (42). Our analysis highlights the disproportionate impacts of global Hg pollution on subsistence fisheries in developing countries, including many island nations far from primary source regions. Unlike seafood consumers that rely on the commercial market and have many food choices, subsistence fishing populations have little control over the type and quantity of seafood they catch and lack other high-quality food alternatives (43). This issue underscores the urgency of global reductions in anthropogenic Hg releases to reduce marine pollution in the future.

## Materials and Methods

### Fisheries Catch Data.

United Nations Food and Agriculture Organization (FAO) catch data provide coarse spatial resolution analysis of MeHg in seafood harvests (20), and past studies have suggested that they may underestimate total global catch due to unreported data on small-scale and illegal fisheries (11). The *Sea Around Us* database combines landings data from the FAO, scientific literature, technical reports, and expert and local knowledge and presents unique fisheries and fisheries-related data at fine spatial scales (14). Our analysis is based on gridded 0.5° × 0.5° resolution catch reconstruction data from the *Sea Around Us* database (11, 14) for 1,774 seafood species that accounts for unreported catches in FAO data (11). We extracted landings data for global industrial, artisanal, and subsistence fisheries between 2001 and 2010 (14). Industrial catches include large amounts of forage fish that are used for fishmeal and fish oil and we separated these species from human consumption estimates based on data from prior work (38). Edible weights of harvests for each seafood category were estimated from live weight landings using conversion factors from prior work (*SI Appendix*, Table S6). For each fishing region (0.5° × 0.5° grid cell) and seafood category, we calculated a catch-weighted mean edible biomass caught by industrial fishing fleets for direct human consumption. All catches by artisanal and subsistence fishing fleets were assumed to be for direct human consumption.

### Mean Global Seafood Mercury Concentrations.

Existing biological monitoring data for MeHg in fish are insufficient to support a comprehensive global analysis of seafood MeHg concentrations (23, 44). Error propagation in model simulations across media (seawater to fish) produces large uncertainties in fish mercury concentrations. To address this challenge, we constrained differences in mercury concentrations among trophic levels (TLs) using empirically measured data (means and ranges) in commercial seafood from Karimi et al. (13). We chose this dataset because it comprehensively synthesized the grand mean of mercury concentrations in commercially important marine fish from diverse government monitoring programs and scientific studies. The database provides information on 90 categories of globally harvested commercial seafood across different TLs (bivalves, prey fish, omnivores, and piscivores), which we use to represent the grand mean global concentration in this work. Mercury concentrations in many commercially important lower TL marine species are limited or not available. Representative geographic coverage of the 90 categories of seafood in the Karimi et al. (13) database is also limited, so we used a modeling approach based on fisheries ecology and seawater MeHg concentrations to derive regional variability within empirical ranges.

MeHg is the only form of mercury that biomagnifies in food webs and typically accounts for >90% of the total mercury in predatory fish (45). We converted total mercury concentrations (13) to MeHg based on measured averages from past work (shellfish = 40% and finfish = 95%) (*SI Appendix*, Table S6) (45–47). For species known to be especially important for MeHg exposure (e.g., tunas, billfishes, salmon), either due to high concentrations or frequent consumption, we based global mean MeHg values on measured total mercury concentrations and the conversions to MeHg above (*SI Appendix*, Table S6).

Since TL explains much of the variability in seafood MeHg concentrations due to efficient biomagnification (48), we developed a statistical relationship between these two factors (*SI Appendix*, Fig. S6 and Table S7, $\log_{10}MeHg=0.51\times TL+0.14$; R^2^ = 0.73). We used this empirical relationship and TL data from the *Sea Around Us* database to estimate global mean MeHg concentrations for missing seafood categories. Using data on species ecology and TL, we aggregated species into four categories: 1) filter feeders (TL: 2.00 to 2.30; MeHg: 0.014 to 0.021 μg/g wet weight); 2) planktivorous and omnivorous fish (TL: 2.31 to 3.50; MeHg: 0.021 to 0.084 μg/g wet weight); 3) piscivorous fish (TL: 3.51 to 4.39; MeHg: 0.085 to 0.24 μg/g wet weight); and 4) large predators (TL: 4.40 to 5.00, MeHg: 0.24 to 0.49 μg/g wet weight) (*SI Appendix*, Table S6). This categorization is not a strict ecological definition but is useful for simplifying results and visualizing data. For each seafood category, we calculated a global mean MeHg concentration from the catch-weighted concentrations of each species included. Factors known to affect seafood MeHg concentrations such as foraging depth (49, 50) and environmental exposures (MeHg concentrations in seawater) (19) were accounted for during regional scaling of global mean concentrations.

### Regional Seafood MeHg Concentrations.

To account for regional variability in MeHg across harvesting locations, we scaled global mean MeHg concentrations for each seafood category within their empirically defined ranges by variability in seawater MeHg concentrations at their respective harvesting locations and feeding depths. Seawater MeHg concentrations were from prior work using the Massachusetts Institute of Technology general circulation model (MITgcm) (19). Modeling seawater MeHg concentrations is necessary because global seawater measurements are relatively sparse and unavailable in many important fishing regions (19). The MITgcm has a resolution of 1° × 1° horizontally and 50 vertical levels. The resolution is higher near the equator (0.5° latitude × 1° longitude) and the Arctic (~40 × 40 km). The model represents average open-ocean concentrations for the same period as seafood harvesting data in this study (19). In prior work, the model was evaluated against available seawater MeHg measurements and showed no statistically significant difference between modeled and measured seawater MeHg concentrations in the surface and subsurface ocean (19).

Modeled seawater MeHg concentrations are not reliable for the continental margin and shelf areas because fine-scale variability in the hydrodynamics of near-shore ecosystems, including potentially polluted continental discharges, are not captured by the relatively coarse (1° × 1°) resolution of the MITgcm (19). Spatial averaging in the model can lead to underestimates of seawater MeHg concentrations in near-shore regions. We therefore synthesized measured MeHg concentrations on the shelf and in marginal seas and scaled modeled concentrations by empirically derived values (*SI Appendix*, Table S8). We excluded literature values from small enclosed or semienclosed coastal ecosystems (e.g., lagoons, bays) strongly influenced by point sources because most commercial fishing activity is concentrated on the shelf and in marginal seas. The coastal scaling is restricted to regions with observational data. Future studies that produce additional empirical MeHg data, particularly in coastal waters predicted to have high seawater MeHg (e.g., west coast of Africa), will be valuable for refining understanding of MeHg concentrations in fisheries from these coastal regions.

We calculated spatially resolved MeHg concentrations within each seafood category ($S_{i}$, Eq. **1**) as follows:

[1] $$S_{i}\left(x,y\right)=\omega_{i}\delta_{i}\left(x,y\right),$$

where $\left(x,y\right)$ represent the capture locations (latitude, longitude) of seafood category *i*; $\delta_{i}$ is the spatial scaling factor for seafood category *i* based on the seawater MeHg concentration at the capture location and feeding depth (Eq. **2**); and $\omega_{i}$ is the scaling factor for each seafood category that constrains the catch-weighted mean to be equal to the empirically derived global value ${(E}_{i}^{\mathrm{mean}})$ (Eq. **3**).

The spatial scaling factor ($\delta_{i}$) for each seafood category was calculated as follows:

[2] $$\delta_{i}\left(x,y\right)=\frac{W_{z}\left(x,y\right)}{{W_{z}}^{\max}}\times E_{i}^{max-min}+E_{i}^{\min},$$

where $W_{z}$ is the seawater MeHg concentration at location $\left(x,y\right)$ averaged over the foraging depth range (*z*) of seafood category *i*.

${W_{z}}^{\max}$ is the maximum seawater MeHg concentration across fished locations. $E_{i}^{max-min}$ represents the empirical range of MeHg concentration in seafood category *i*, and $E_{i}^{\min}$ is the minimum empirical MeHg concentration in seafood category *i.*

In this way, variability in MeHg concentrations within each seafood category is constrained to be equal to the empirically measured dynamic range ($\frac{E_{i}^{\max}}{E_{i}^{\min}}$) reported in prior work (13). $\omega_{i}$ was chosen for each seafood category such that the empirically derived global mean MeHg concentration for each seafood category $\left(E_{i}^{\mathrm{mean}}\right)$ was equal to the regionally modeled catch-weighted mean:

[3] $$E_{i}^{\mathrm{mean}}=\frac{\sum_{\left(x,y\right)}S_{i}\left(x,y\right)\times C_{i}\left(x,y\right)}{\sum_{\left(x,y\right)}C_{i}\left(x,y\right)},$$

where *S_i_* is the spatially resolved MeHg concentration, defined above, and *C_i_* is the catch of species (*i*).

This spatial scaling method constrains simulated MeHg concentrations based on their measured means and ranges for each seafood category. Relative seawater MeHg concentrations are used to spatially scale seafood Hg concentrations across their empirically constrained values, while the global mean seafood MeHg concentrations are set to the empirically determined values. The total mass of MeHg fished is therefore insensitive to the absolute value of seawater MeHg concentrations.

*SI Appendix* describes uncertainty and sensitivity analysis for results using the IQR of measured Hg concentrations for each seafood category (*SI Appendix*, Table S6). We also consider how the spatial distribution of seawater MeHg concentrations influences the global pattern and budget of MeHg extracted by marine fisheries (*SI Appendix*, section 1 and Figs. S2 and S3).

### Model Evaluation and Sensitivity Analysis.

We compiled empirical yellowfin tuna MeHg data for diverse migratory cohorts (*n* = 1,482 from 24 ocean regions) that had statistically significant differences in MeHg concentrations (*SI Appendix*, Table S1) (51–53). The authors of prior work linked variability in Hg concentrations to trophic status and size but did not account for differences in seawater MeHg concentrations (51, 52). The market size for yellowfin tuna is typically 5 to 20 kg (9). We used the relationship between fork length and MeHg concentrations characterized in prior work (51) to normalize observed MeHg concentrations to a common size (90 cm or 15 kg) within the common market size range (5 to 20 kg) (*SI Appendix*, Fig S1 and Table S1). We excluded juveniles (<4 kg) because their dietary composition and TL differ from adults and they are smaller than the typical size in the commercial market. Changes in model performance based on different assumptions for coastal seawater MeHg scaling are shown in *SI Appendix* and do not result in major differences (*SI Appendix*, section 1 and Figs. S4 and S5).

For all individual seafood categories, we simulated the vertical range of MeHg exposures using data from Fishbase.com on feeding depths (*SI Appendix*, Table S2). Some large pelagic fish horizontally migrate beyond the spatial scale of the modeled grid cell used to simulate seawater MeHg (1° latitude × 1° longitude). We assessed the impacts of horizontal migration for regionally migrating species like yellowfin tuna for up to 500 km from the harvest location by scaling fish MeHg concentrations based on modeled seawater concentrations from a broader horizontal range. Accounting for broader horizontal seawater scaling only produced minor changes in our results (*SI Appendix*, Fig. S7 and Table S9). Given higher computational expenses for simulating horizontal migration and only small improvements in predictive performance, we chose to use the native seawater MeHg model for spatial scaling of seafood categories in this study.

### Global MeHg Fished from Oceans.

We calculated the mass flows of MeHg from global marine fisheries into the commercial seafood market for human consumption. For each 0.5° latitude × 0.5° longitude ocean grid cell, we extracted the sum product of all category-specific MeHg concentrations and corresponding edible catches to calculate the total MeHg mass extracted by fisheries at that location. The total MeHg mass from marine fisheries was further categorized based on fisheries composition, harvest location, fishing sector (i.e., subsistence, artisanal, industrial), and fishing entity using data from the *Sea Around Us* database (14). The amounts of MeHg fished at any location reflect the product of weighted MeHg concentrations for each seafood type caught at that location and the biomass of each type harvested (Fig. 1).

### MeHg Exposures for Subsistence Fishers.

Small-scale and non-commercial fishing activities that produce seafood to feed the families of fishers and/or local communities are defined as *subsistence fishing* in the *Sea Around Us* database (14). Biomonitoring data from coastal and island communities that rely heavily on seafood for subsistence show some of the highest levels of MeHg exposure globally (10). Based on the regional catches and corresponding MeHg concentrations flowing into each subsistence fishing location, we estimated how much seafood can be consumed before exceeding regulatory exposure limits. These calculations included scenarios for MeHg exposure based on the U.S. EPA RfD (0.1 μg per kg BW per day) (54, 55). We compared subsistence MeHg exposures to the U.S. EPA RfD rather than other international values because it provides a margin of safety in the dose–response relationship for MeHg among sensitive groups by applying an uncertainty factor of 10 (12).

We used the *Sea Around Us* data (14) on subsistence fishing catches to calculate a catch-weighted mean MeHg concentration for each region with a recorded subsistence population globally (173 entities). We estimated the quantity of fish that could be consumed before exceeding established exposure limits for each subsistence fishing population using the mean MeHg concentration for the mixture of seafood caught in the region (*C_MeHg_*, μg g^−1^) and probabilistically simulated BWs (kg) drawn from a regionally specific distribution (i.e., *CR*, g day^body weights (BW1^ = *RfD ×*BW/*C*_MeHg_) (56, 57). Adult BWs were assumed to be normally distributed (58) and means and SD for each subsistence population were obtained from published data (59) (*SI Appendix*, Table S10).

### Mapping MeHg and Nutrients in EEZ.

We estimated the ratios of MeHg-to-micronutrients (omega-3 fatty acids and selenium: Se) in capture fisheries for 242 EEZs for fisheries globally (sovereign state exclusive fishing regions). Specifically, we calculated catch-weighted mean MeHg concentration for each EEZ using category-specific MeHg concentrations and corresponding edible catches in each EEZ (Eq. **4**). Prior work (30) simulated micronutrient concentrations in seafood species harvested in each EEZ using Bayesian hierarchical models and empirical measurements of nutrients and species-level environmental and ecological traits. Our study was conducted for a comparable time as the prior work on micronutrients and used the same fisheries catch data. We calculated micronutrient-to-MeHg ratios based on spatially averaged, catch-weighted EEZ MeHg concentrations and EEZ-specific nutrient concentrations. Reliable databases on micronutrients in seafood are rapidly evolving, and this analysis could be updated when future advances in understanding seafood nutrients are mature (60).

[4] $${\mathrm{MeHg}}_{EEZ-mean}=\frac{\sum_{i}\sum_{\left(x,y\right)}S_{i}\left(x,y\right)\times C_{i}\left(x,y\right)}{\sum_{i}\sum_{\left(x,y\right)}C_{i}\left(x,y\right)},$$

where *S_i_* $\left(x,y\right)$ and *C_i_* $\left(x,y\right)$ are the spatially resolved MeHg concentration and catches of seafood category *i* at location $\left(x,y\right)$ within a given EEZ, respectively. Therefore, the numerator represents the summed MeHg fished over all seafood categories and locations within a given EEZ, and the denominator is the total edible catches from the EEZ.

## Supplementary Material

Appendix 01 (PDF)

## Data Availability

All model code and data will be made available on GitHub (https://github.com/SunderlandLab/global_fisheries_hg) (61) and Dataverse (https://doi.org/10.7910/DVN/KVC2WH) (62). All other data are included in the article and/or *SI Appendix*.
