# Supplementary material for: Global fishing patterns amplify human exposures to methylmercury
Source: Proc Natl Acad Sci U S A. 2024 Sep 23;121(40):e2405898121. doi: 10.1073/pnas.2405898121 (PMC11459155; doi:10.1073/pnas.2405898121)
Supplement: Supplementary file 1 — Appendix 01 (PDF) [file pnas.2405898121.sapp.pdf]

Supporting Information for

Global Fishing Patterns Amplify Human Exposures to Methylmercury

Mi-Ling Li[1,2,3*], Colin P. Thackray[2], Vicky W.Y. Lam[3],
William W.L. Cheung[3], Elsie M. Sunderland[2,4]

[1]School of Marine Science and Policy, University of Delaware, Newark, DE 19711, USA.
[2]Harvard John A. Paulson School of Engineering & Applied Sciences, Harvard University, Cambridge, MA, 02138, USA.
[3]Institute for the Oceans and Fisheries, University of British Columbia, Vancouver, BC, V6T 1Z4, Canada.
[4]Department of Environmental Health, Harvard T.H. Chan School of Public Health, Boston MA, 02115, USA.

*Corresponding author: Mi-Ling Li (milingli@udel.edu)

The PDF file includes:

Supporting text: Section 1. Model Uncertainty and Sensitivity Analysis
Figures S1 to S7
Table S1 to S10
SI References

1. Model Uncertainty and Sensitivity Analysis

We used the interquartile range (IQR) of the global mean MeHg concentrations in each seafood category to estimate the uncertainty in global MeHg mass flows from fisheries (SI Table S6). The IQR of the MeHg concentration was calculated directly from empirical data or from the IQR of the regression slope between MeHg concentrations and trophic level derived in this study (SI Fig. S6). Our analysis suggests 6.1 tonnes (IQR: 4.2-9.5 tonnes) of MeHg is removed from the ocean annually by fisheries catches, with 84% in edible biomass intended for direct human consumption (5.1 tonnes, IQR 3.5-7.8 tonnes) and 16% in non-human food such as fishmeal for aquaculture. Results show similar spatial patterns of MeHg fished from the ocean given the IQR variability of the global mean MeHg concentrations categories (Figure S2-S3).

Our best estimate (i.e., baseline simulation) of regional seafood MeHg concentrations is derived from scaling global mean MeHg concentrations for each seafood category within their empirically defined ranges using modeled global seawater MeHg concentrations and an additional scaling factor for coastal seawater (see Methods). We compared our best estimate with model simulations using uniform seawater MeHg concentrations across the global oceans and coastal waters. We find the modeled seawater concentrations do not change the overall spatial pattern of the MeHg mass flows (Fig. S2 and S3). However, our base simulation produces a larger harvest region of overlapping upper quartiles of catch biomass and MeHg concentrations in low-latitude fisheries compared to the scenario that does not scale seafood MeHg concentrations based on variability in seawater MeHg concentrations (Figure S3a-b). These results indicate the important role of elevated seawater MeHg levels in increasing regional MeHg concentrations in the fisheries harvested from these productive tropical and subtropical marine regions.

We scaled modeled MeHg concentrations by empirical values for MeHg in coastal waters, where available, to account for a potential bias in concentrations due to spatial averaging across coarse global model grid cells (Table S8). To assess how the coastal scaling approach influenced modeled MeHg flows from coastal fisheries, we compared results for two different scenarios: (1) No coastal scaling to account for higher MeHg levels in coastal waters observed in empirical studies; (2) 200% coastal scaling factor to amplify coastal seawater MeHg levels. Results shown in Figure S3 indicate that our scaling assumptions have minimal effects on ratios of micronutrients to MeHg in fisheries catches from most EEZs, except for the Mediterranean region (Figure S4).

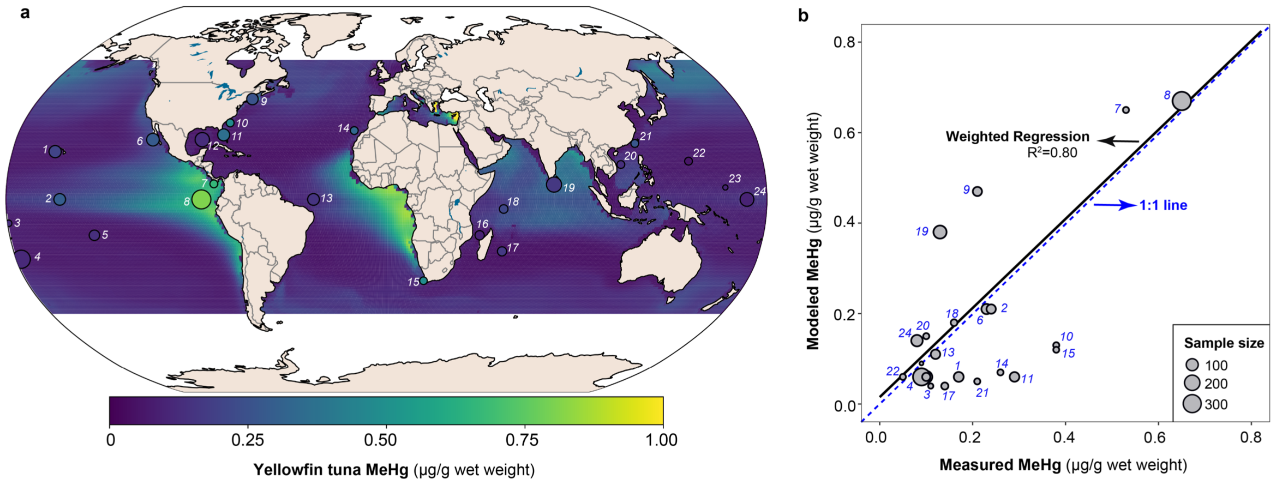

Figure S1. Comparison of empirical and modeled MeHg concentrations in 1482 yellowfin tuna from 24 ocean locations. Panel (**a)** shows modeled yellowfin tuna MeHg concentrations as the background color over its distribution range (59°N - 48°S). Circles show observed MeHg concentrations for size normalized yellowfin (length = 90cm; weight=15kg) (1). Circle size and color represent the sample size of each study and the average MeHg concentration of yellowfin tuna (see data in Table S1). The site number is indicated next to each circle. Panel (**b)** shows a weighted linear regression of measured versus modeled yellowfin tuna MeHg concentration (black line; y=1.0x+0.01; R^2=0.80). The weight is the sample size of each study, indicated by circle size. Site numbers are shown on both panels.

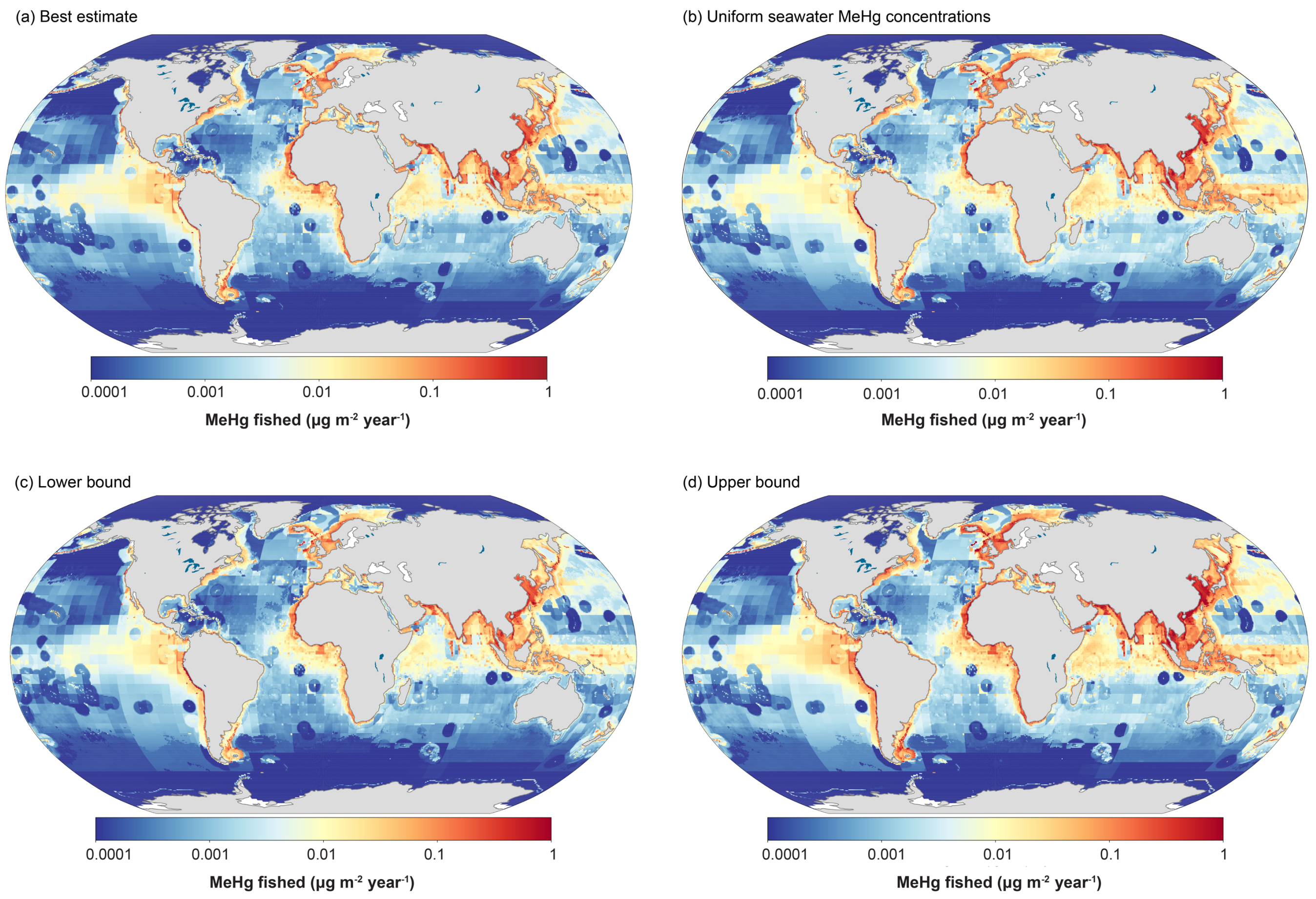

Figure S2. Uncertainty in methylmercury (MeHg) fished from the ocean. Panel (a) shows our best estimate of spatially resolved MeHg mass flows from the ocean annually through seafood harvests (blue = low and red = high), as illustrated in Figure 1. Panel (b) illustrates modeled MeHg mass flows using uniform seawater MeHg across global oceans (i.e., no modeled differences in seawater MeHg concentrations). Panels (c) and (d) show results using the lower and upper bounds of interquartile range of global seafood MeHg concentrations (SI Table S6).

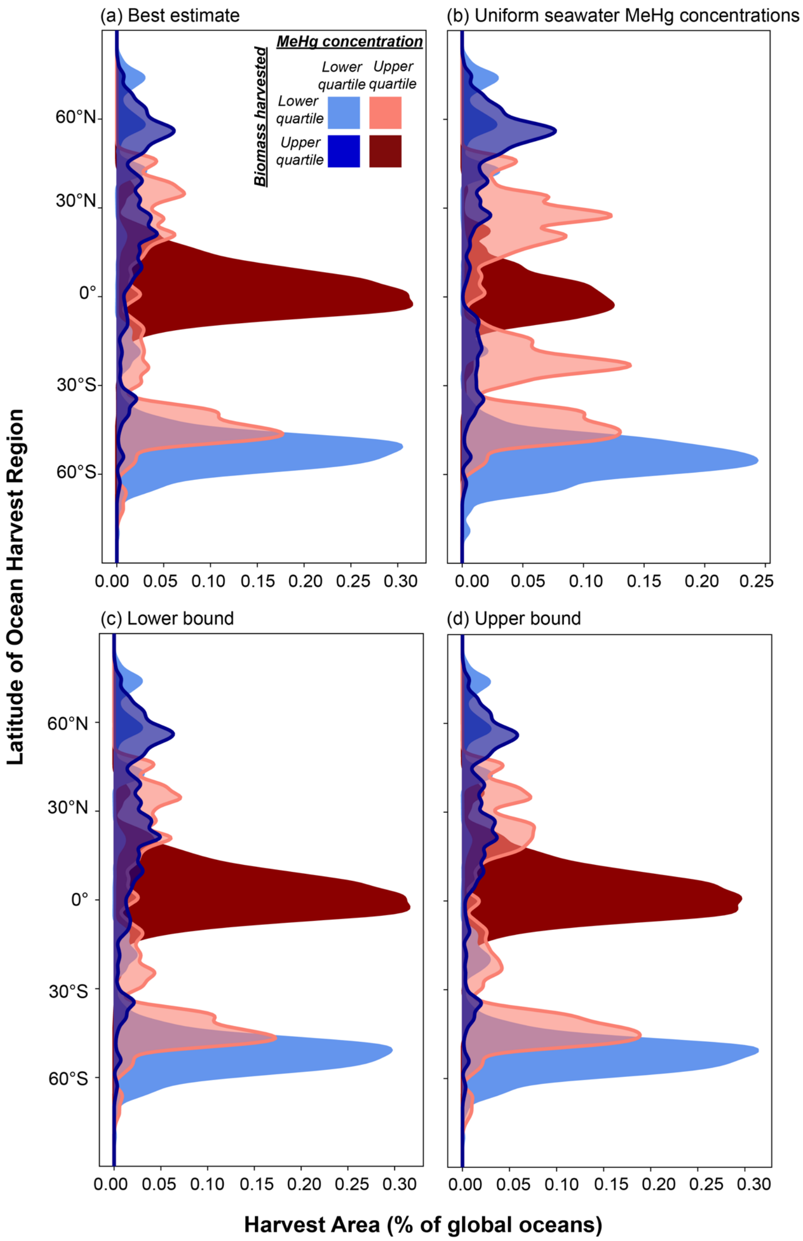

Figure S3. Uncertainty in overlapping seafood biomass harvests and catch-weighted methylmercury (MeHg) concentrations combination. Panel (a) shows our best estimate as illustrated in Figure 2b in the main text. Panel (b) illustrates modeling results with uniform seawater MeHg concentrations (no spatial scaling). Panels (c) and (d) show results using spatial modeling approach with the lower and upper bounds of the interquartile range of global seafood MeHg concentrations (Table S6), respectively. Ocean regions that are highlighted include (i)

high MeHg and high catch (maroon), (ii) high MeHg and low catch (pink), (iii) high catch and low MeHg (dark blue), and (iv) low catch and low MeHg (light blue).

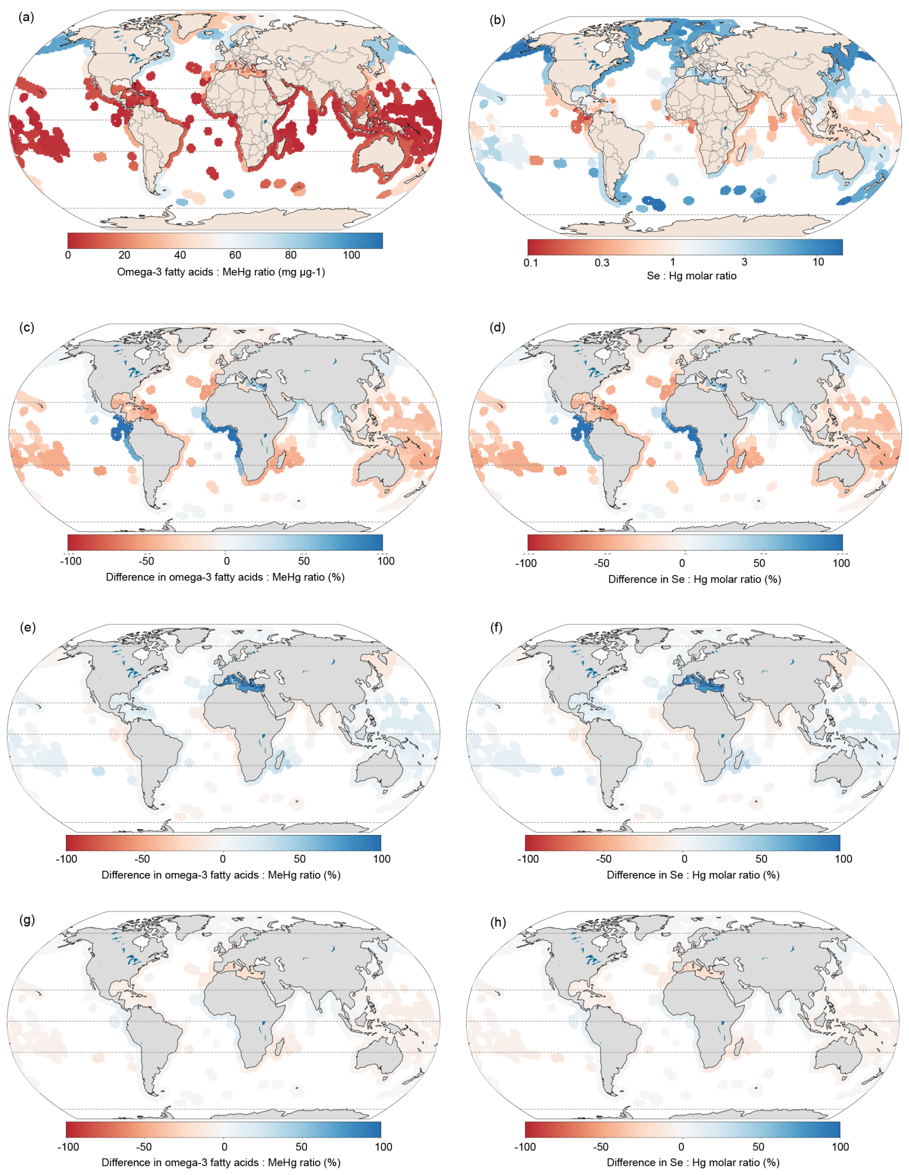

Figure S4. Sensitivity of micronutrients to MeHg ratios in fisheries catches from global EEZs to varying coastal seawater MeHg concentrations. Panels (a-b) illustrate the baseline simulation with average ratios of omega-3 fatty acids and selenium-to-MeHg for the catch-weighted fisheries harvest in each EEZ. The baseline scenario uses modeled global seawater MeHg concentrations from past work (2) and an additional scaling factor for coastal seawater (see Methods), as presented in the main text and Figure 3. Panels (c-h) show the difference between the baseline simulation and scenarios with: (c-d) uniform seawater MeHg concentrations, (e-f) global seawater spatial distribution with no coastal scaling factors and (g-h) doubling of coastal scaling factors used for baseline simulation.

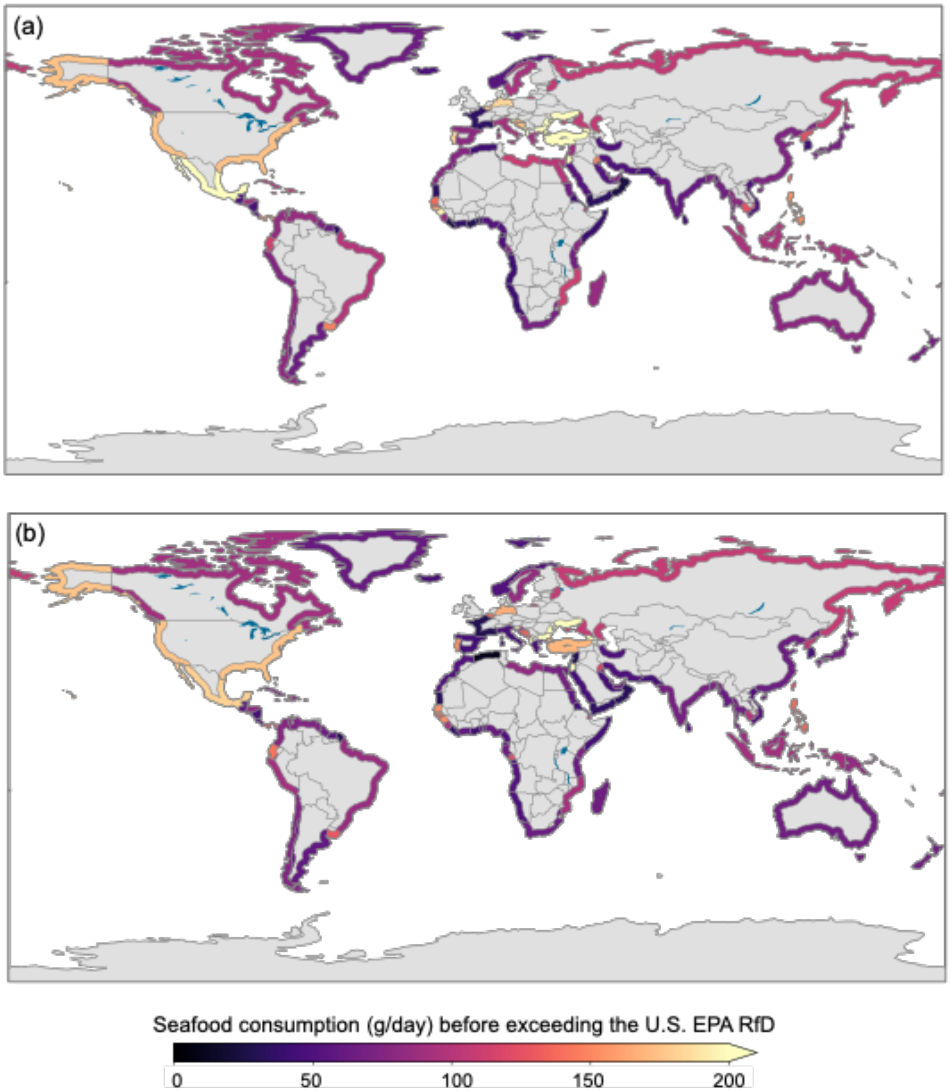

Figure S5. Sensitivity of subsistence fish consumption rates without appreciable risk of MeHg exposure to coastal seawater scaling assumptions. The colored coastal region denote the average g/day of seafood that can be consumed by each of the 173 marine subsistence fishing populations before exceeding the United States Environmental Protection Agency's Reference Dose (U.S. EPA RfD) (0.1 µg/kg per day), based on their catch-weighted methylmercury (MeHg) concentrations (See Methods). Panel (a) shows the average g/day of seafood calculated without scaling MeHg concentrations in coastal areas, and panel (b) shows a doubling of coastal scaling factors used in the baseline simulation.

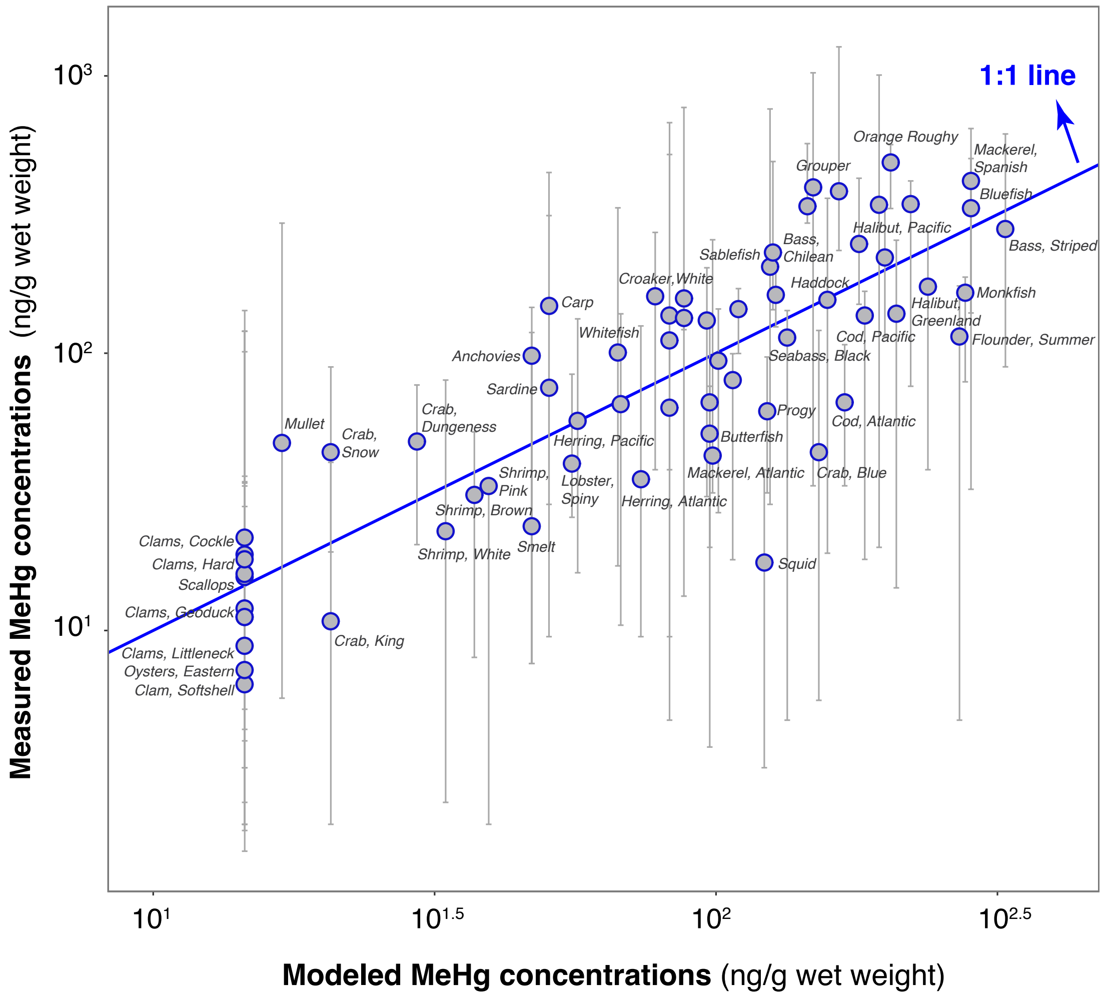

Figure S6. **Comparison of measured and modeled MeHg concentrations for 65 seafood groups**. Measured MeHg concentrations are based on a prior synthesis of commercial market seafood Hg measurements (3). Modeled values are based on an empirically derived relationship with trophic level for each species (SI Table S7). Error bars indicate the minimum and maximum in concentrations reported across published datasets (3). Some seafood names are not shown due to space constraints and all data are available in SI Table S7.

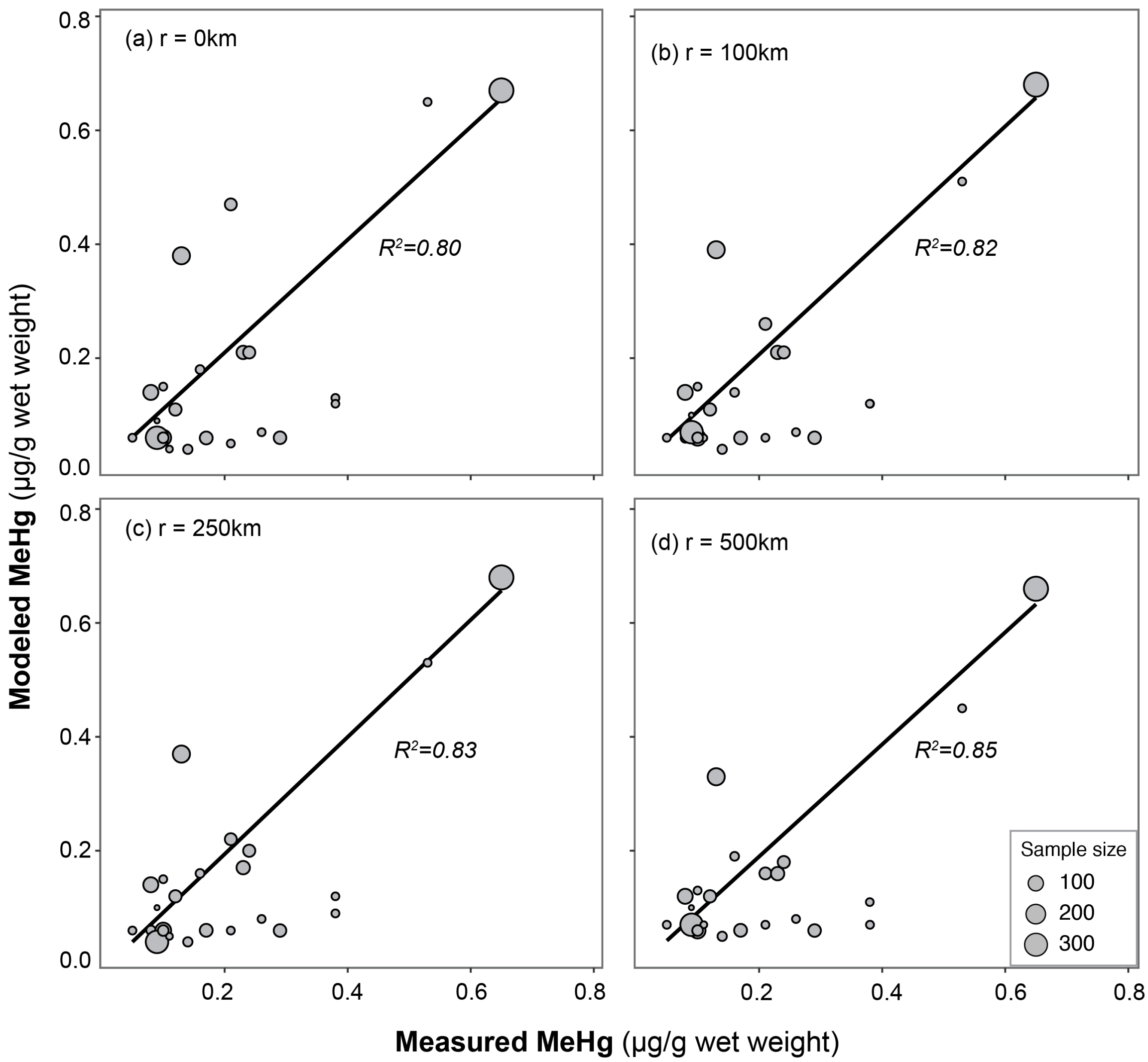

Figure S7. Comparison of model performance for yellowfin tuna MeHg concentrations across 24 ocean regions based on different horizontal migration ranges used to model seawater MeHg exposures. The symbol '*r*' represents the radius from the center of grid cell where harvests occur. The black line shows a weighted linear regression of modeled versus measured yellowfin tuna MeHg concentrations (weight ~15kg). Regressions are weighted by the sample size of each study, indicated by circle sizes. Raw data are available in Table S9.

Table S1. Comparison of simulated and empirical MeHg concentrations (μg/g wet weight) of yellowfin tuna of a common market size (90cm or 15kg) and range (5-20kg) across 24 ocean regions.

Site	Sample size	Longitude [a]	Latitude [a]	Empirical concentration (range) [b]	Modeled concentration [c]
Indian (Reunion Island)	19	55	-22	0.14 (0.05, 0.17)	0.04
Indian (Mozambique channel)	20	44	-15	0.08 (0.03, 0.09)	0.06
Indian	15	55	-4	0.16 (0.06, 0.21)	0.18
Indian (Sri Lanka)	140	79	6	0.13 (0.04, 0.16)	0.38
Atlantic (New England)	47	-70	42	0.21 (0.07, 0.26)	0.47
Atlantic (North and South Carolina)	10	-78	32	0.38 (0.14, 0.47)	0.13
Atlantic (Northeast)	10	-16	29	0.26 (0.09, 0.33)	0.07
Atlantic (Florida)	56	-80	27	0.29 (0.10, 0.36)	0.06
Atlantic (Southeast)	9	18	-34	0.38 (0.14, 0.48)	0.12
Atlantic (Brazil)	52	-35	0	0.12 (0.04, 0.15)	0.11
Atlantic (Gulf of Mexico)	112	-90	25	0.10 (0.04, 0.13)	0.06
Pacific (South China Sea)	10	111	15	0.10 (0.04 ,0.13)	0.15
Pacific (North China Sea)	10	120	23	0.21 (0.07, 0.26)	0.05
Pacific (Hawaii)	60	-160	20	0.17 (0.06, 0.21)	0.06
Pacific (Northwest)	10	144	16	0.05 (0.02, 0.06)	0.06
Pacific (ARCHm/WCPO)	292	-178	-25	0.09 (0.03, 0.12)	0.06
Pacific (NPTG/WCPO)	2	160	5	0.09 (0.03, 0.11)	0.09
Pacific (PEQD/WCPO)	5	180	-10	0.11 (0.04, 0.13)	0.04
Pacific (SPSGm/WCPO)	32	-140	-15	0.10 (0.04, 0.13)	0.06
Pacific (WARMm/WCPO)	96	170	0	0.08 (0.03, 0.10)	0.14
Pacific (Ecuador) [d]	347	-88	0	0.65 (0.23, 0.81)	0.67
Pacific (California)	68	-114	25	0.23 (0.08, 0.28)	0.21
Pacific (Central Equatorial)	50	-155	0	0.24 (0.08, 0.30)	0.21
Pacific (East)	10	-82	7	0.53 (0.19, 0.66)	0.65

a. If not explicitly included in the publication, capture location is estimated based on the description of the study site.
b. Normalized observed MeHg concentrations to a common size (90cm or 15kg) and range (62-98cm or 5-20kg) (6) based on the relationship between tuna length and muscle total Hg concentration described in Houssard et al., 2019 (1) and the assumption that 95% of total Hg is MeHg in finfish (see Methods). Empirical concentrations of yellowfin tuna in various sizes across global locations were compiled by Houssard et al., 2019 (1) unless stated otherwise.
c. Modeled MeHg concentration based on the average seawater concentration at the corresponding feeding depths (1-250m) of the capture location.
d. Data come from Munoz Abril 2016 (20).

Table S2. Fisheries catches and MeHg contribution in each spatial category described in Figure 2.

Spatial pattern	MeHg category	Catch (tonnes/year)	MeHg (g/year)
High MeHg-High Catch	Large Pelagics	1.67E+06	8.62E+05
	Large Predators	8.92E+04	2.57E+04
	Piscivores	2.41E+05	4.71E+04
	Planktivores & Omnivores	6.49E+05	8.17E+04
	Filter Feeders	4.73E+03	8.40E+01
	Others	2.13E+03	1.85E+03
High MeHg-Low Catch	Large Pelagics	5.53E+03	2.20E+03
	Large Predators	3.49E+02	1.04E+02
	Piscivores	4.64E+02	8.20E+01
	Planktivores & Omnivores	1.03E+03	1.21E+02
	Filter Feeders	6.00E+00	0.00E+00
	Others	0.00E+00	0.00E+00
Low MeHg-High Catch	Large Pelagics	1.65E+05	1.75E+04
	Large Predators	5.08E+04	1.55E+04
	Piscivores	7.40E+05	1.19E+05
	Planktivores & Omnivores	5.94E+06	3.95E+05
	Filter Feeders	2.44E+05	6.38E+03
	Others	1.03E+06	3.72E+04
Low MeHg-Low Catch	Large Pelagics	2.17E+02	1.01E+02
	Large Predators	2.00E+00	1.00E+00
	Piscivores	7.80E+01	1.30E+01
	Planktivores & Omnivores	1.13E+04	1.28E+03
	Filter Feeders	2.14E+02	4.00E+00
	Others	4.60E+01	1.00E+00

Table S3. The concentration (μg/g wet weight) of MeHg and micronutrients in fisheries catches in global EEZs.

EEZ	Se	Omega-3 fatty acids	MeHg
Albania	0.116	3567	0.285
Algeria	0.117	3346	0.111
Andaman & Nicobar Isl. (India)	0.064	441	0.456
Angola	0.071	2138	0.290
Anguilla (UK)	0.022	355	0.128
Antigua & Barbuda	0.014	19	0.128
Argentina	0.293	7519	0.115
Aruba (Netherlands)	0.066	1002	0.140
Ascension Isl. (UK)	0.167	850	0.492
Australia	0.134	1303	0.114
Azores Isl. (Portugal)	0.140	861	0.261
Bahamas	0.050	80	0.129
Bahrain	0.019	196	0.045
Balearic Island (Spain)	0.174	3213	0.187
Bangladesh	0.067	1277	0.172
Barbados	0.085	565	0.140
Belgium	0.155	2726	0.057
Belize	0.077	350	0.094
Benin	0.033	755	0.313
Bermuda (UK)	0.057	437	0.216
Bonaire (Netherlands)	0.057	186	0.134
Bouvet Isl. (Norway)	0.188	5158	0.059
Brazil	0.077	1949	0.146
British Virgin Isl. (UK)	0.047	70	0.127
Brunei Darussalam	0.016	108	0.129
Cambodia	0.023	439	0.082
Cameroon	0.021	214	0.071
Canada (East Coast)	0.256	6197	0.084
Canada (Pacific)	0.230	3803	0.057
Canary Isl. (Spain)	0.124	279	0.211
Cape Verde	0.067	437	0.355
Chagos Archipelago (UK)	0.045	36	0.236
Channel Isl. (UK)	0.163	3950	0.073
Chile	0.091	4661	0.080
China	0.147	2559	0.085
Christmas Isl. (Australia)	0.049	21	0.226
Clipperton Isl. (France)	0.067	24	0.427
Colombia (Caribbean)	0.021	244	0.118
Colombia (Pacific)	0.043	440	0.432
Comoros Isl.	0.038	381	0.142
Congo (ex-Zaire)	0.138	2208	0.217
Congo R. of	0.090	2776	0.566
Cook Islands	0.151	388	0.118
Corsica (France)	0.206	3400	0.196
Costa Rica (Caribbean)	0.059	212	0.096
Costa Rica (Pacific)	0.050	296	0.794
Côte d'Ivoire	0.083	1463	0.342
Crete (Greece)	0.210	3393	0.095
Croatia	0.082	4457	0.119
Crozet Isl. (France)	0.362	1746	0.092

Cuba	0.046	741	0.083
Curaçao (Netherlands)	0.070	441	0.100
Cyprus (North)	0.271	445	0.311
Cyprus (South)	0.201	3521	0.133
Denmark (Baltic Sea)	0.197	4563	0.049
Denmark (North Sea)	0.189	5242	0.053
Desventuradas Isl. (Chile)	0.218	1467	0.241
Djibouti	0.016	373	0.122
Dominica	0.074	33	0.142
Dominican Republic	0.020	98	0.130
Easter Isl. (Chile)	0.262	2511	0.117
Ecuador	0.067	1537	0.497
Egypt (Mediterranean)	0.136	2485	0.094
Egypt (Red Sea)	0.023	1411	0.068
El Salvador	0.042	0	0.522
Equatorial Guinea	0.033	93	0.414
Eritrea	0.025	1706	0.070
Faeroe Isl. (Denmark)	0.257	7173	0.084
Falkland Isl. (UK)	0.284	7290	0.115
Brazil (Fernando de Noronha)	0.145	423	0.333
Fiji	0.064	279	0.146
France (Atlantic Coast)	0.219	4872	0.073
France (Mediterranean)	0.186	4792	0.129
French Guiana	0.035	281	0.096
French Polynesia	0.121	365	0.186
Gabon	0.146	2322	0.377
Galapagos Isl. (Ecuador)	0.048	8	0.646
Gambia	0.116	1225	0.209
Germany (North Sea)	0.136	5753	0.051
Ghana	0.090	2737	0.542
Glorieuse Islands (France)	0.045	34	0.136
Greece	0.169	3532	0.171
Greenland	0.274	2944	0.083
Grenada	0.075	666	0.159
Guadeloupe (France)	0.039	2	0.118
Guam (USA)	0.040	634	0.183
Guatemala (Pacific)	0.027	0	0.518
Guinea	0.077	1162	0.189
Guinea-Bissau	0.187	2478	0.271
Guyana	0.103	1155	0.160
Haiti	0.058	696	0.104
Hawaii Main Islands (USA)	0.195	1647	0.206
Hawaii Northwest Islands (USA)	0.205	1587	0.209
Heard & McDonald Isl. (Australia)	0.360	2588	0.083
Honduras (Caribbean)	0.052	492	0.077
Howland & Baker Isl. (USA)	0.045	3	0.192
Iceland	0.208	6989	0.079
India (mainland)	0.070	2148	0.207
Indonesia (Central)	0.032	453	0.074
Indonesia (Eastern)	0.026	375	0.099
Indonesia (Indian Ocean)	0.036	631	0.173
Iran (Persian Gulf)	0.063	1038	0.100
Iran (Sea of Oman)	0.046	573	0.307
Ireland	0.250	5724	0.098

Israel (Mediterranean)	0.132	1704	0.163
Italy	0.145	4684	0.136
Jamaica	0.031	350	0.102
Jan Mayen Isl. (Norway)	0.261	2203	0.059
Japan (Daito Islands)	0.135	328	0.154
Japan (Ogasawara Islands)	0.138	539	0.160
Japan (main islands)	0.178	4978	0.106
Jarvis Isl. (USA)	0.060	17	0.237
Johnston Atoll (USA)	0.171	109	0.109
Juan Fernandez Islands (Chile)	0.178	805	0.063
Kenya	0.026	153	0.164
Kerguelen Isl. (France)	0.364	1644	0.123
Kermadec Isl. (New Zealand)	0.248	1048	0.158
Kiribati (Gilbert Islands)	0.044	5	0.167
Kiribati (Line Islands)	0.106	125	0.214
Kiribati (Phoenix Islands)	0.045	7	0.167
Korea (North)	0.345	7032	0.085
Korea (South)	0.159	3177	0.086
Kuwait	0.101	852	0.078
Lebanon	0.079	1057	0.452
Liberia	0.101	1367	0.335
Libya	0.182	2542	0.082
Lord Howe Isl. (Australia)	0.111	1213	0.186
Macquarie Isl. (Australia)	0.385	1230	0.067
Madagascar	0.028	478	0.127
Madeira Isl. (Portugal)	0.203	4660	0.202
Malaysia (Peninsula East)	0.027	902	0.074
Malaysia (Peninsula West)	0.031	910	0.083
Malaysia (Sabah)	0.037	1105	0.117
Malaysia (Sarawak)	0.034	1230	0.081
Maldives	0.036	82	0.294
Malta	0.178	1119	0.240
Marshall Isl.	0.052	14	0.189
Martinique (France)	0.03	139	0.142
Mauritania	0.122	3060	0.168
Mauritius	0.066	122	0.204
Mayotte (France)	0.036	87	0.127
Mexico (Atlantic)	0.058	1218	0.097
Mexico (Pacific)	0.065	2409	0.302
Micronesia (Federated States of)	0.044	4	0.169
Montenegro	0.151	2699	0.243
Montserrat (UK)	0.030	381	0.159
Morocco (Central)	0.087	4215	0.093
Morocco (Mediterranean)	0.167	4183	0.166
Morocco (South)	0.098	4210	0.095
Mozambique	0.021	1044	0.151
Mozambique Channel Isl. (France)	0.059	91	0.191
Myanmar	0.051	1000	0.280
Namibia	0.142	5331	0.507
Nauru	0.046	4	0.161
Netherlands	0.157	4357	0.059
New Caledonia (France)	0.147	396	0.170
New Zealand	0.357	5596	0.132
Nicaragua (Caribbean)	0.035	120	0.075

Nicaragua (Pacific)	0.036	12	0.274
Nigeria	0.037	1354	0.339
Niue (New Zealand)	0.161	440	0.133
Norfolk Isl. (Australia)	0.213	776	0.147
Northern Marianas (USA)	0.101	928	0.186
Norway	0.244	5504	0.080
Oman	0.032	705	0.250
Oman (Musandam)	0.019	447	0.118
Pakistan	0.067	1222	0.501
Palau	0.054	59	0.144
Palmyra Atoll & Kingman Reef (USA)	0.090	37	0.271
Panama (Caribbean)	0.064	171	0.145
Panama (Pacific)	0.027	883	0.659
Papua New Guinea	0.041	5	0.140
Peru	0.06	5710	0.209
Philippines	0.021	909	0.079
Portugal	0.138	4362	0.152
South Africa (Prince Edward Islands)	0.388	1040	0.095
Puerto Rico (USA)	0.037	671	0.140
Qatar	0.025	205	0.086
Reunion (France)	0.162	675	0.250
Russia (Barents Sea)	0.246	5655	0.104
Russia (Far East)	0.379	7683	0.092
Saba & Sint Eustaius (Netherlands)	0.070	80	0.158
Saint Helena (UK)	0.154	329	0.287
Saint Kitts & Nevis	0.034	171	0.088
Saint Lucia	0.037	149	0.176
Saint Pierre & Miquelon (France)	0.284	1218	0.077
Saint Vincent & the Grenadines	0.071	573	0.193
Samoa	0.094	535	0.141
Sao Tome & Principe	0.066	323	0.560
Sardinia (Italy)	0.161	3042	0.094
Saudi Arabia (Persian Gulf)	0.027	133	0.091
Saudi Arabia (Red Sea)	0.025	323	0.080
Senegal	0.196	1876	0.261
Seychelles	0.057	25	0.230
Sicily (Italy)	0.173	3928	0.119
Sierra Leone	0.027	600	0.379
Solomon Isl.	0.053	72	0.145
Somalia	0.03	225	0.150
South Africa (Atlantic Coast)	0.102	5553	0.177
South Africa (Indian Ocean Coast)	0.180	813	0.142
South Georgia & Sandwich Isl. (UK)	0.359	2622	0.060
South Orkney Islands (UK)	0.222	9097	0.104
Spain (Northwest)	0.213	5061	0.087
Spain (Mediterranean and Gulf of Cadiz)	0.127	4762	0.149
Sri Lanka	0.036	755	0.264
Brazil (St Paul and St. Peter Archipelago)	0.136	572	0.506
Suriname	0.061	972	0.122
Svalbard Isl. (Norway)	0.269	3081	0.070
Sweden (West Coast)	0.204	5670	0.064
Syria	0.080	1707	0.502

Taiwan	0.122	3539	0.121
Tanzania	0.027	619	0.141
Thailand (Andaman Sea)	0.026	933	0.200
Thailand (Gulf of Thailand)	0.027	958	0.076
Timor Leste	0.016	263	0.130
Togo	0.079	3032	0.319
Tokelau (New Zealand)	0.048	27	0.138
Tonga	0.110	417	0.117
Trindade & Martim Vaz Isl. (Brazil)	0.179	880	0.231
Trinidad & Tobago	0.022	220	0.129
Tristan da Cunha Isl. (UK)	0.206	514	0.229
Tromelin Isl. (France)	0.181	517	0.175
Tunisia	0.161	2659	0.083
Turkey (Mediterranean Sea)	0.097	3292	0.219
Turks & Caicos Isl. (UK)	0.188	1031	0.128
Tuvalu	0.044	10	0.144
USA (Alaska Subarctic)	0.397	6487	0.067
USA (East Coast)	0.255	5521	0.079
USA (Gulf of Mexico)	0.069	2643	0.049
USA (West Coast)	0.176	4884	0.101
US Virgin Islands	0.025	36	0.073
United Arab Emirates	0.024	267	0.162
United Arab Emirates (Fujairah)	0.032	604	0.095
United Kingdom	0.208	5796	0.090
Uruguay	0.240	5444	0.133
Vanuatu	0.143	435	0.147
Venezuela	0.112	2247	0.118
Vietnam	0.066	1277	0.111
Wake Isl. (USA)	0.170	317	0.176
Wallis & Futuna Isl. (France)	0.077	266	0.144
Yemen (Arabian Sea)	0.027	888	0.214
Yemen (Red Sea)	0.052	1012	0.075

Table S4. The seafood MeHg concentration in subsistence catches and fish consumption rates before exceeding US EPA reference dose in global subsistence populations.

		Fish consumption rates before exceeding US EPA reference dose (g/day)					
**Fishing Entity**	**MeHg concentration (µg/g wet weight)**	**mean**	**5th percentil e**	**25th percentil e**	**50th percentil e**	**75th percentile**	**95th percentile**
Albania	0.01	701	469	607	701	795	932
Algeria	0.45	14	9	12	14	15	18
American Samoa	0.11	62	42	54	62	71	83
Angola	0.13	48	32	41	48	54	63
Anguilla (UK)	0.06	104	69	90	104	119	139
Antigua & Barbuda	0.06	105	70	91	105	119	140
Argentina	0.11	60	40	52	60	68	80
Aruba (Netherlands)	0.13	53	35	46	53	61	71
Australia	0.10	71	48	62	71	81	94
Azores Isl. (Portugal)	0.08	82	55	71	82	93	109
Bahamas	0.05	126	84	109	126	143	168
Bahrain	0.05	128	85	111	128	145	170
Bangladesh	0.08	73	49	63	73	82	96
Barbados	0.08	84	57	73	84	96	111
Belgium	0.05	153	102	132	153	173	204
Belize	0.12	57	39	50	57	65	76
Benin	0.12	52	34	45	52	59	69
Bermuda (UK)	0.16	42	28	37	42	48	56
Bonaire (Netherlands)	0.11	63	42	55	63	72	84
Bosnia & Herzegovina	0.05	151	101	131	151	172	201
Brazil	0.08	90	61	78	90	102	120
British Virgin Isl. (UK)	0.08	87	59	76	87	99	116
Brunei Darussalam	0.06	95	63	82	95	108	126
Cambodia	0.05	108	73	93	107	122	143
Cameroon	0.12	51	34	44	51	58	68
Canada	0.09	91	61	79	92	104	122
Cape Verde	0.12	58	39	51	58	66	77
Cayman Isl. (UK)	0.14	50	34	44	50	57	67
Chile	0.09	77	52	67	77	87	102
China	0.09	67	45	58	67	76	89
Christmas Isl. (Australia)	0.09	74	49	64	74	84	98
Cocos (Keeling) Isl. (Australia)	0.10	68	46	59	68	78	91
Colombia	0.09	80	53	69	80	90	106
Comoros	0.08	83	55	71	83	94	110
Congo (ex-Zaire)	0.14	44	30	38	44	50	59
Congo R. of	0.06	98	66	85	98	111	130
Cook Islands	0.09	74	50	64	74	84	99
Costa Rica	0.14	50	33	43	50	56	66
Cote d'Ivoire	0.12	50	33	43	50	56	66
Croatia	0.07	107	71	92	106	121	142
Cuba	0.08	82	55	71	82	93	109
Curaçao	0.09	74	49	64	73	83	98
Djibouti	0.06	98	66	85	98	112	131
Dominica	0.10	67	45	58	67	76	89
Dominican Republic	0.08	86	58	74	86	98	115
Ecuador	0.05	129	87	112	129	146	172
Egypt	0.08	73	49	63	73	83	98

El Salvador	0.08	87	58	76	88	99	115
Equatorial Guinea	0.15	40	26	34	40	45	53
Eritrea	0.14	43	29	38	43	49	57
Faeroe Isl. (Denmark)	0.13	53	36	46	53	60	70
Falkland Isl. (UK)	0.06	115	78	100	115	131	153
Fiji	0.08	87	59	75	87	99	116
France	0.09	78	52	68	78	89	104
French Guiana	0.21	32	21	28	32	36	42
French Polynesia	0.09	75	50	65	75	84	99
Gabon	0.10	60	40	51	60	68	79
Gambia	0.04	167	112	145	167	190	223
Gaza Strip	0.09	66	44	57	66	75	88
Germany	0.04	169	114	146	169	192	224
Ghana	0.17	35	24	30	35	40	47
Greece	0.14	50	33	43	50	56	66
Greenland	0.13	62	41	54	62	70	83
Grenada	0.12	55	37	48	55	62	73
Guadeloupe (France)	0.08	80	53	70	81	91	107
Guam (USA)	0.10	70	47	60	69	79	92
Guatemala	0.13	53	36	46	53	60	70
Guinea	0.04	169	113	146	169	192	224
Guinea-Bissau	0.04	146	97	126	146	165	193
Guyana	0.10	68	45	59	68	77	90
Haiti	0.06	110	74	95	110	124	145
Honduras	0.08	84	57	73	84	95	112
Iceland	0.15	47	32	41	47	54	63
India	0.09	66	44	57	66	75	88
Indonesia	0.08	92	62	80	92	104	122
Iran	0.12	49	33	43	49	56	65
Iraq	0.05	127	85	110	127	144	168
Israel	0.17	34	23	30	34	39	46
Italy	0.13	54	37	47	54	61	72
Jamaica	0.09	75	50	65	75	85	99
Japan	0.08	68	46	59	68	77	91
Kenya	0.08	74	50	64	74	84	98
Kiribati	0.13	52	35	45	52	59	69
Korea (North)	0.05	110	74	95	110	125	147
Korea (South)	0.13	45	30	39	45	51	59
Kuwait	0.08	73	49	64	73	84	98
Lebanon	0.31	19	12	16	19	21	24
Liberia	0.18	34	23	29	34	38	45
Libya	0.07	89	59	77	89	101	118
Madagascar	0.08	72	48	62	72	81	95
Madeira Isl. (Portugal)	0.08	89	60	77	89	101	118
Malaysia	0.08	90	60	78	90	102	120
Maldives	0.22	32	21	27	32	36	42
Malta	0.20	36	24	31	36	41	48
Marshall Isl.	0.09	80	54	69	80	91	106
Martinique (France)	0.07	92	62	80	92	104	122
Mauritania	0.18	33	22	29	33	38	44
Mauritius	0.06	107	71	92	107	121	142
Mayotte (France)	0.11	53	36	46	53	61	71
Mexico	0.04	189	128	164	190	215	250
Micronesia	0.08	83	56	72	84	95	111
Montenegro	0.05	154	103	134	154	175	205
Montserrat (UK)	0.06	115	77	100	116	131	154
Morocco	0.10	58	39	51	58	66	78

Mozambique	0.06	100	67	86	100	113	132
Myanmar	0.09	64	43	55	64	73	85
Namibia	0.13	46	31	40	46	52	61
Nauru	0.13	51	34	44	51	59	69
New Caledonia (France)	0.10	67	45	58	66	76	88
New Zealand	0.11	67	45	58	67	76	89
Nicaragua	0.15	46	31	40	46	52	61
Nigeria	0.10	59	39	51	59	67	79
Niue (New Zealand)	0.07	100	67	86	100	114	133
North Cyprus	0.20	36	24	31	36	40	47
North Marianas (USA)	0.12	59	39	51	59	67	79
Norway	0.14	51	34	44	51	58	68
Oman	0.20	28	19	25	28	32	38
Pakistan	0.13	45	30	39	45	51	60
Palau	0.10	70	47	61	70	79	93
Panama	0.05	140	94	120	140	158	185
Papua New Guinea	0.09	85	57	74	86	97	114
Peru	0.08	86	58	74	85	97	114
Philippines	0.05	145	98	126	145	164	193
Pitcairn (UK)	0.05	146	98	127	146	166	194
Portugal	0.04	164	111	142	164	187	218
Puerto Rico (USA)	0.08	90	60	78	90	103	120
Qatar	0.10	60	40	52	60	68	80
Russian Federation	0.07	107	72	92	107	121	142
Saba and Saint Eustaius (Netherlands)	0.06	114	76	99	114	129	151
Saint Kitts & Nevis	0.06	108	73	94	108	123	144
Saint Lucia	0.08	90	61	78	90	102	120
Saint Pierre & Miquelon (France)	0.05	137	93	119	137	155	182
Saint Vincent & the Grenadines	0.11	63	42	54	62	71	83
Samoa	0.10	68	46	59	68	77	90
Sao Tome & Principe	0.48	14	10	12	14	16	19
Saudi Arabia	0.12	47	31	41	47	53	62
Senegal	0.04	147	98	127	147	166	195
Sierra Leone	0.07	90	60	78	90	102	120
Singapore	0.05	107	72	92	107	121	141
Sint Maarten	0.06	118	79	102	118	134	156
Slovenia	0.02	380	255	328	380	432	507
Solomon Isl.	0.10	67	45	58	67	76	89
Somalia	0.14	43	29	37	43	49	57
South Africa	0.09	65	44	57	65	74	87
South Cyprus	0.23	30	20	26	30	34	40
Spain	0.14	51	35	44	51	58	68
Sri Lanka	0.12	49	33	43	49	56	66
St Barthelemy (France)	0.09	78	52	67	78	88	104
St Martin	0.09	78	53	68	78	89	104
Sudan	0.10	60	40	52	60	68	80
Suriname	0.12	56	38	49	56	64	75
Sweden	0.08	86	57	75	86	98	115
Syrian Arab Republic	0.18	32	21	28	32	36	42
Taiwan	0.05	128	86	111	128	145	170
Tanzania	0.09	65	43	56	65	73	86
Thailand	0.08	70	47	61	70	80	94
Timor Leste	0.12	63	42	55	63	72	84
Togo	0.17	41	27	35	41	46	54

Tokelau (New Zealand)	0.09	78	52	68	78	89	104
Tonga	0.05	147	98	127	147	167	196
Trinidad & Tobago	0.11	61	41	52	60	69	81
Turkey	0.03	228	153	196	228	258	303
Turks & Caicos Isl. (UK)	0.05	137	92	119	137	155	182
Tuvalu	0.06	120	81	103	120	136	159
United Arab Emirates	0.09	67	45	58	67	76	89
Uruguay	0.05	135	91	117	136	153	180
US Virgin Isl.	0.06	111	74	96	110	126	148
USA	0.05	178	120	154	178	202	236
Vanuatu	0.06	119	80	103	119	135	159
Venezuela	0.09	78	52	68	78	89	104
Viet Nam	0.11	51	34	44	51	58	68
Wallis & Futuna Isl. (France)	0.08	84	56	72	84	95	111
Yemen	0.17	35	23	30	35	39	46

Table S5. The MeHg contribution (in %) of each seafood category in subsistence catches.

Fishing Entity	Filter Feeders	Planktivores & Omnivores	Piscivores	Large Predators	Large Pelagics	Others
Albania	2	84	13	1	0	0
Algeria	0	5	7	5	84	0
American Samoa	1	12	87	0	1	0
Angola	0	40	26	5	29	0
Anguilla (UK)	0	55	42	2	1	0
Antigua & Barbuda	0	55	42	0	2	0
Argentina	0	14	86	0	0	0
Aruba (Netherlands)	0	9	79	13	0	0
Australia	1	24	39	1	36	0
Azores Isl. (Portugal)	0	39	58	3	0	0
Bahamas	1	75	24	1	0	0
Bahrain	0	100	0	0	0	0
Bangladesh	1	49	50	0	0	0
Barbados	1	36	44	0	19	0
Belgium	0	100	0	0	0	0
Belize	0	13	76	10	0	1
Benin	0	74	26	0	0	0
Bermuda (UK)	0	5	14	67	13	0
Bonaire (Netherlands)	0	26	23	39	11	0
Bosnia & Herzegovina	0	94	2	0	4	0
Brazil	0	44	52	0	3	0
British Virgin Isl. (UK)	0	37	62	0	0	0
Brunei Darussalam	0	62	3	0	35	0
Cambodia	0	77	23	0	0	0
Cameroon	0	18	57	9	15	0
Canada	0	14	50	23	0	13
Cape Verde	0	32	59	9	0	0
Cayman Isl. (UK)	0	3	93	5	0	0
Chile	0	37	35	28	0	0
China	1	35	32	30	2	0
Christmas Isl. (Australia)	0	39	61	0	0	0
Cocos (Keeling) Isl. (Australia)	1	22	77	0	0	0
Colombia	0	36	61	0	3	0
Comoros	0	36	10	0	53	0
Congo (ex-Zaire)	0	26	30	0	43	0
Congo R. of	0	100	0	0	0	0
Cook Islands	1	10	18	0	71	0
Costa Rica	0	5	95	0	0	0
Croatia	0	81	8	0	11	0
Cote d'Ivoire	0	60	40	0	0	0
Cuba	0	34	51	0	15	0
Curacao	0	37	20	34	10	0
Djibouti	0	100	0	0	0	0
Dominica	0	26	15	31	7	21
Dominican Republic	0	33	46	3	18	0
Ecuador	0	85	5	2	8	0
Egypt	1	45	54	0	0	0
El Salvador	0	56	44	0	0	0
Equatorial Guinea	0	15	85	0	0	0
Eritrea	0	7	65	15	14	0
Faeroe Isl. (Denmark)	0	8	92	0	0	0
Falkland Isl. (UK)	0	100	0	0	0	0
Fiji	2	33	56	9	0	0

France	0	26	73	0	0	0
French Guiana	0	4	28	0	68	0
French Polynesia	0	25	72	2	1	0
Gabon	0	40	52	0	9	0
Gambia	3	97	0	0	0	0
Gaza Strip	0	63	35	2	0	0
Germany	31	0	69	0	0	0
Ghana	0	39	7	0	53	0
Greece	0	38	59	0	2	0
Greenland	0	2	97	0	0	0
Grenada	0	7	93	0	0	0
Guadeloupe (France)	0	32	66	0	2	0
Guam (USA)	1	21	69	0	9	0
Guatemala	0	25	53	4	19	0
Guinea	0	100	0	0	0	0
Guinea-Bissau	4	86	10	0	0	0
Guyana	0	27	52	8	13	0
Haiti	0	60	39	1	0	0
Honduras	1	47	25	9	6	13
Iceland	0	1	89	9	0	0
India	1	51	42	7	0	0
Indonesia	0	42	47	0	11	0
Iran	0	18	57	26	0	0
Iraq	0	100	0	0	0	0
Israel	0	31	30	0	38	0
Italy	0	25	36	7	32	0
Jamaica	0	27	64	0	8	0
Japan	1	23	44	11	14	7
Jordan	0	32	19	6	42	0
Kenya	0	44	43	2	9	0
Kiribati	1	11	31	0	58	0
Korea (North)	2	74	24	0	0	0
Korea (South)	1	5	95	0	0	0
Kuwait	0	37	46	0	17	0
Lebanon	0	26	22	0	52	0
Liberia	0	32	34	1	33	0
Libya	0	59	26	15	0	0
Madagascar	0	33	36	10	19	0
Madeira Isl. (Portugal)	0	41	57	2	0	0
Malaysia	0	37	50	3	10	0
Maldives	0	0	14	0	86	0
Malta	0	7	12	68	13	0
Marshall Isl.	1	25	58	0	16	0
Martinique (France)	0	42	58	0	0	0
Mauritania	0	11	29	7	53	0
Mauritius	0	58	42	0	0	0
Mayotte (France)	0	8	47	7	37	0
Mexico	0	100	0	0	0	0
Micronesia	4	24	66	6	0	0
Montenegro	0	82	10	0	9	0
Montserrat (UK)	1	65	26	4	1	2
Morocco	1	30	29	16	26	0
Mozambique	0	64	33	0	2	0
Myanmar	0	20	63	0	17	0
Namibia	0	17	83	0	0	0
Nauru	0	8	30	0	63	0
New Caledonia (France)	0	20	74	6	0	0

New Zealand	0	15	56	19	6	3
Nicaragua	0	5	77	4	15	0
Nigeria	0	37	63	0	0	0
Niue (New Zealand)	1	47	18	4	30	0
North Cyprus	0	53	38	10	0	0
North Marianas (USA)	0	10	16	11	62	0
Norway	0	3	92	6	0	0
Oman	0	16	18	0	66	0
Pakistan	0	11	60	27	2	0
Palau	2	17	82	0	0	0
Panama	0	93	7	0	0	0
Papua New Guinea	1	29	62	0	8	0
Peru	1	54	44	0	0	0
Philippines	0	100	0	0	0	0
Pitcairn (UK)	0	100	0	0	0	0
Portugal	1	64	35	0	0	0
Puerto Rico (USA)	0	37	49	9	6	0
Qatar	0	22	78	0	0	0
Russian Federation	0	33	23	0	0	44
Saba and Saint Eustaius (Netherlands)	0	63	37	0	0	0
Saint Kitts & Nevis	0	61	26	12	1	0
Saint Lucia	1	38	58	0	3	0
Saint Pierre & Miquelon (France)	0	100	0	0	0	0
Saint Vincent & the Grenadines	0	14	82	1	2	0
Samoa	0	20	68	1	11	0
Sao Tome & Principe	0	10	9	5	77	0
Saudi Arabia	0	9	60	29	0	0
Senegal	10	90	0	0	0	0
Sierra Leone	0	83	16	0	1	0
Singapore	1	73	20	6	0	0
Sint Maarten	0	67	33	0	0	0
Slovenia	0	88	1	0	11	0
Solomon Isl.	0	20	79	0	2	0
Somalia	0	11	67	0	21	0
South Africa	1	26	74	0	0	0
South Cyprus	0	36	22	5	37	0
Spain	0	24	50	6	20	0
Sri Lanka	0	41	24	4	30	0
St Barthelemy (France)	1	27	49	13	10	0
St Martin	1	27	49	13	10	0
Sudan	0	20	80	0	0	0
Suriname	0	10	83	7	1	0
Sweden	0	16	84	0	0	0
Syrian Arab Republic	0	65	35	0	0	0
Taiwan	4	96	0	0	0	0
Tanzania	1	26	67	0	6	0
Thailand	0	34	62	2	2	0
Timor Leste	0	19	22	2	57	0
Togo	0	22	78	0	0	0
Tokelau (New Zealand)	0	38	10	16	36	0
Tonga	13	53	34	1	0	0
Trinidad & Tobago	0	8	44	3	46	0
Turkey	0	19	37	0	44	0
Turks & Caicos Isl. (UK)	0	88	7	2	2	0
Tuvalu	0	64	3	0	33	0

United Arab Emirates	0	29	70	0	1	0
Uruguay	0	96	4	0	0	0
US Virgin Isl.	0	60	29	10	1	0
USA	0	16	15	20	0	48
Vanuatu	1	69	28	0	2	0
Venezuela	1	27	53	11	9	0
Viet Nam	0	18	64	0	19	0
Wallis & Futuna Isl. (France)	1	35	54	9	0	0
Yemen	0	23	6	4	67	0

Table S6. Summary of fish methylmercury (MeHg) categories.

MeHg category	No. of species included	Major fish composition	Mean MeHg conc. (IQR) (μg/g w.w.) [a]	Mean tropic level [g]	Edible weight (% of live weight) [h]	Assigned feeding depth (m) [j]	Major habitat
Filter feeders	210	Bivalves, clupeidae	0.016 (0.012-0.021) [b]	2.08	20	0-50	Coastal
Planktivores & omnivores	611	Anchovy, crab, shrimp	0.055 (0.039-0.084) [b]	3.09	60	0-200	Coastal/Oceanic
Piscivores	710	Pompanos, whiting, cod	0.15 (0.095-0.249) [b]	3.93	47 [i]	0-1000	Coastal/Oceanic
Large predators	109	Hairtail, large Scombridae	0.26 (0.162-0.474) [b]	4.46	47 [i]	0-1000	Coastal/Oceanic
Albacore tuna	1	Albacore tuna	0.30 (0.23-0.37)	4.30	58	0-600	Oceanic
Bigeye tuna	1	Bigeye tuna	0.55 (0.41-0.69)	4.49	58	0-1500	Oceanic
Skipjack tuna	1	Skipjack tuna	0.19 (0.14-0.24)	4.43	62	0-260	Oceanic
Yellowfin tuna	1	Yellowfin tuna	0.26 (0.18-0.34)	4.41	58	1-250	Oceanic
Other tunas	12	All other tuna	0.21 (0.13-0.38) [b]	4.29	60	10-200	Coastal/Oceanic
Blue marlin	3	Blue marlin	2.34 (0.99-3.70)	4.47	60	0-1000	Oceanic
Other Billfishes	12	Swordfish, Other marlin species,	0.85 (0.66-1.04) [c]	4.52	60	0-2827	Oceanic
King mackerel	1	King Mackerel	1.05 (0.81-1.29)	4.42	67	5-140	Coastal
Pollock	1	Alaska Pollock	0.048 (0.031-0.067)	3.57	41	0-1280	Coastal
Salmon	8	All salmon species	0.046 (0.031-0.061) [d]	4.12	62	0-250	Coastal
Shark_high	67	Shark with trophic level ≥ 4	0.84 (0.55-1.13) [e]	4.19	44	1-1000	Oceanic
Shark_low	20	Shark with trophic level < 4	0.26 (0.22-0.30) [f]	3.74	44	20-195	Coastal

a. Mean and interquartile range (IQR) of MeHg concentrations are calculated from total Hg concentrations and standard errors in Karimi, et al. except otherwise noted.
b. Catch weighted mean MeHg concentrations are predicted based on trophic levels and the IQR is calculated based the IQR of the regression slope (see Methods).
c. Converted from total Hg concentrations of swordfish in Karimi, et al. (3).
d. Averaged MeHg concentrations of all salmon species included in Karimi, et al. (3).
e. Calculated based on the average of grand mean total Hg concentrations of blue, blacktip, mako, sandbar, and thresher sharks included in Karimi, et al. (3).
f. Dogfish concentration from Taylor et al. (4) is used to represent Shark_low category.
g. Catch weighted mean trophic level based on data from *SeaAroundUs* (5).
h. Edible conversion factors from Sunderland except otherwise noted (6).
i. Conversion factor of 'White fish generally' is used (7).
j. Feeding depth is the full depth range from FishBase (8). Species that have the largest catches in the category are used to represent the feeding depth range and major feeding habitat of categories that encompass many species – frigate and bullet tuna, swordfish, pink salmon, blue shark, and narrownose smooth-hound/blacktip reef shark represent Other tunas, Billfish, Salmon, Shark_high, and Shark_low, respectively.

Table S7. Synthesized global mean MeHg concentrations (ng/g wet weight) in 65 seafood categories and the range of mean reported MeHg concentrations across studies.

Seafood Item	Mean MeHg [a]	Min [a]	Max [a]	Trophic Level (TL) [b]	Representative species for estimating TL
Anchovies	97.9	7.6	146.3	3.00	
Bass, Chilean	339.2	294.5	570.0	3.96	Patagonian toothfish
Bass, Striped	280.3	89.3	617.5	4.65	
Bluefish	333.5	32.3	646.0	4.53	
Butterfish	51.3	3.8	76.0	3.62	
Carp	148.2	28.5	448.4	3.06	Common carp
Catfish	136.8	4.8	678.3	3.48	Siluriformes
Clams (All)	11.2	2.0	120.0	2.00	
Clams, Cockle	21.6	7.6	101.2	2.00	Common cockle
Clams, Geoduck	12.0	4.0	16.4	2.00	
Clams, Hard	18.8	2.0	120.0	2.00	
Clams, Pacific Littleneck	8.8	4.4	11.2	2.00	Veneridae
Clams, Softshell	6.4	3.2	34.4	2.00	Sand gaper
Cod, Atlantic	66.5	33.3	107.4	4.09	
Cod, Pacific	136.8	18.1	167.2	4.16	
Crab, Blue	44.0	5.6	120.8	4.00	
Crab, Dungeness	48.0	20.4	76.8	2.60	
Crab, King	10.8	2.0	40.4	2.30	
Crab, Snow	44.0	19.2	89.2	2.30	
Croaker, Atlantic	65.6	10.5	138.7	3.31	
Croaker, White	160.6	38.0	272.7	3.43	
Eel	205.2	28.5	760.0	3.83	American eel
Flounder, Summer	115.0	4.8	174.8	4.49	
Flounder, Windowpane	144.4	99.8	171.0	3.72	
Flounder, Winter	66.5	20.0	138.7	3.62	
Freshwater Perch	134.0	13.3	769.5	3.53	Perch-like Perciformes
Grouper	396.2	33.3	1026.0	3.98	Basses, groupers, hinds
Haddock	155.8	19.0	362.0	4.03	
Hake	138.7	14.3	255.6	4.27	
Halibut, Greenland	173.9	38.0	274.6	4.38	
Halibut, Pacific	248.0	150.1	427.5	4.14	
Herring, Atlantic	35.2	9.5	125.4	3.38	
Herring, Pacific	57.0	16.2	133.0	3.16	
Lingcod	344.9	76.0	418.0	4.32	
Lobster, American	80.0	18.0	99.6	3.70	
Lobster, Spiny	40.0	25.6	84.0	3.14	
Mackerel, Atlantic	42.8	31.4	256.5	3.63	
Mackerel, Chub	94.1	26.6	144.4	3.65	

Mackerel, Spanish	418.0	139.7	503.5	4.53	Atlantic Spanish mackerel
Monkfish	165.3	78.9	188.1	4.51	Lophius americanus
Mullet	47.5	5.7	294.5	2.13	Flathead grey mullet
Mussels	11.2	5.2	34.0	2.00	Blue mussels
Ocean Perch	111.2	9.5	520.6	3.48	
Orange Roughy	487.4	332.5	565.3	4.25	
Oysters, Eastern	7.2	2.4	33.2	2.00	American cupped oyster
Oysters, Pacific	15.6	3.2	28.0	2.00	Pacific cupped oyster
Pike	383.8	234.7	1273.0	4.07	
Porgy	61.8	31.4	96.9	3.82	Scup
Sablefish	230.9	143.5	491.2	3.84	
Sardine	75.1	9.5	313.5	3.06	Sardina pilchardus
Scallops	16.0	1.6	36.0	2.00	
Seabass, Black	114.0	4.8	142.5	3.89	
Shad, American	63.7	38.0	105.5	3.48	
Sheepshead	157.7	121.6	187.2	3.53	
Shrimp, Brown	30.8	8.0	52.0	2.80	Average of the northern and southern species
Shrimp, Pink	33.2	2.0	34.0	2.85	
Shrimp, White	22.8	2.4	80.0	2.70	
Skate	131.1	30.4	203.3	3.61	Little skate
Smelt	23.8	7.6	118.8	3.00	Rainbow smelt
Snapper, Gray	221.4	115.0	330.6	4.23	Grey snapper
Squid	17.6	3.2	38.0	3.81	Squids, cuttlefishes, octopuses
Tilapia	18.1	1.9	142.5	3.85	Great northern tilefish
Tilefish, Atlantic	162.5	124.5	217.6	3.00	
Weakfish/Seatrout	343.0	20.0	1007.0	4.21	Weakfishes
Whitefish	100.7	17.1	334.4	3.30	

a. Derived from total Hg data in Karimi, et al. 2012 (3). Derived from total Hg data to MeHg concentrations based on measured average fractions from past work (shellfish = 40% and finfish = 95%) (9–11).

b. Data from *Sea Around Us* databases (5).

Table S8. Observed and simulated values of MeHg concentrations (pM) in coastal waters.

Location	Observed range	Observed average MeHg	Simulated range	Simulated average MeHg	Depth (m)	References
San Francisco Bay[a]	0-1.6	NA	0.01-0.4	0.18	~0.5	Conaway et al., 2003(12)
North Sea (Belgian coastal zone)[a]	0-0.25	NA	0.02	0.02	0-10	Leemakers et al., 2001(13)
Mediterranean Sea (Eastern basin) [a,c]	0.15-0.20	0.17	0.02-0.72	0.17	0-4000	Horvat et al., 2003(14)
Mediterranean Sea (Western basin) [a,c]	0.16-0.26	0.20	0.02-0.61	0.20	0-3000	Horvat et al., 2003(14)
Labrador Sea [b]	0.03-0.12	0.09	0.03-0.31	0.20	0-500	Wang et al., 2018(15)
East China Sea [b]	0-3.20	1.16	0.02-0.08	0.04	0-20	Liu et al., 2020(16)
East Japan Sea (Ulleung Basin) [b]	0.01	0.01	0.01-0.48	0.12	0-300	Yang et al., 2017(17)
East Japan Sea (Ulleung Basin) [b]	0.46-0.50	0.49	0.44-0.51	0.48	300-1000	Yang et al., 2017(17)
East Japan Sea (Japan Basin) [b]	0.01-0.05	0.03	0.01-0.19	0.03	0-200	Yang et al., 2017(17)
East Japan Sea (Japan Basin) [b]	0.32-0.51	0.44	0.31-0.48	0.42	200-1000	Yang et al., 2017(17)
N. Atlantic coast (Rhode Island to North Carolina) [b,c]	0.02-0.10	0.05	0.04-0.06	0.05	0-1000	2017 cruise data (EN596) Northwest Atlantic coast and shelf [d]
N. Atlantic coast (North Carolina to Florida) [b,c]	0.01-0.61	0.13	0.02-0.29	0.09	0-1000	2017 cruise data (EN597) Northwest Atlantic coast and shelf [e]

a. Observed and simulated values are seawater monomethylmercury concentrations.
b. Observed and simulated values are seawater total methylmercury concentrations.
c. To reasonably match synthesized observation data, simulated seawater MeHg concentrations were multiplied by scaling factors of 4 (N. Atlantic coast), 30 (Mediterranean Sea eastern basin), and 10 (Mediterranean western basin).
d. Data collected from surface to 1000 m depth samples at four Northwestern Atlantic shelf stations occupied on RV Endeavor cruise from April 25-29, 2017 (shown as stations in yellow in figure below). Analytical methods for MeHg at Harvard University followed Schartup et al. (2015) (18).
e. Data collected from surface to 1000 m depth samples at nine Northwestern Atlantic coastal and shelf stations occupied on RV Endeavor cruise from April 30-May 5, 2017 (shown as stations in red in figure below). Analytical methods for MeHg at Harvard University followed Schartup et al. (2015) (18).

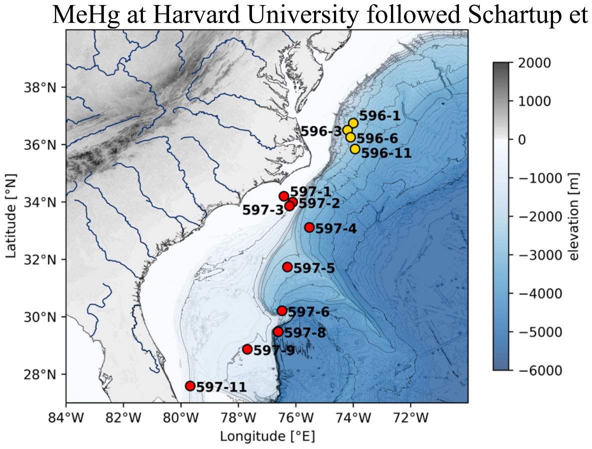

Table S9. Simulated MeHg concentrations (μg/g wet weight) in yellowfin tuna using varying horizontal migration ranges for seawater MeHg exposures.

Site	Empirical MeHg Concentration	Modeled MeHg Concentration			
		r^* = 0 (at harvest location)	r =100km	r =250km	r =500km
Indian (Reunion Island)	0.14	0.04	0.04	0.04	0.05
Indian (Mozambique channel)	0.08	0.06	0.06	0.06	0.07
Indian	0.16	0.18	0.14	0.16	0.19
Indian (Sri Lanka)	0.13	0.38	0.39	0.37	0.33
Atlantic (New England)	0.21	0.47	0.26	0.22	0.16
Atlantic (North and South Carolina)	0.38	0.13	0.12	0.09	0.07
Atlantic (Northeast)	0.26	0.07	0.07	0.08	0.08
Atlantic (Florida)	0.29	0.06	0.06	0.06	0.06
Atlantic (Southeast)	0.38	0.12	0.12	0.12	0.11
Atlantic (Brazil)	0.12	0.11	0.11	0.12	0.12
Atlantic (Gulf of Mexico)	0.10	0.06	0.06	0.06	0.06
Pacific (South China Sea)	0.10	0.15	0.15	0.15	0.13
Pacific (North China Sea)	0.21	0.05	0.06	0.06	0.07
Pacific (Hawaii)	0.17	0.06	0.06	0.06	0.06
Pacific (Northwest)	0.05	0.06	0.06	0.06	0.07
Pacific (ARCHm/WCPO)	0.09	0.06	0.07	0.04	0.07
Pacific (NPTG/WCPO)	0.09	0.09	0.10	0.10	0.10
Pacific (PEQD/WCPO)	0.11	0.04	0.05	0.05	0.07
Pacific (SPSGm/WCPO)	0.10	0.06	0.06	0.06	0.06
Pacific (WARMm/WCPO)	0.08	0.14	0.14	0.14	0.12
Pacific (Ecuador)	0.65	0.67	0.68	0.68	0.66
Pacific (California)	0.23	0.21	0.21	0.17	0.16
Pacific (Central equatorial)	0.24	0.21	0.21	0.2	0.18
Pacific (East)	0.53	0.65	0.51	0.53	0.45

$^*r$ denotes the radius from the center of grid cell where harvest occurs.

Table S10. Body mass by World Health Organization (WHO) region.

WHO region	Average body mass (kg) [a]	Standard Deviation (kg) [b]
Asia	57.7	11.5
Europe	70.8	14.2
Africa	60.7	12.1
Latin America and Caribbean	67.9	13.6
Northern America	80.7	16.1
Oceania	74.1	14.8
Small Islands [c]	68.7	13.7

a. Data from Walpole et al., 2012 (21).
b. Calculated by coefficient variance (0.20) multiplied by average body mass. The coefficient variance is estimated using the distribution of the body weight of 20+ years old U.S. population (22).
c. The mean and standard deviation is calculated by averaging all other regions.

References

1. P. Houssard, *et al.*, A Model of Mercury Distribution in Tuna from the Western and Central Pacific Ocean: Influence of Physiology, Ecology and Environmental Factors. *Env. Sci. Technol.* **53**, 1422–1431 (2019).
2. Y. Zhang, A. L. Soerensen, A. T. Schartup, E. M. Sunderland, A Global Model for Methylmercury Formation and Uptake at the Base of Marine Food Webs. *Global Biogeochem. Cycles* **34**, e2019GB006348 (2020).
3. R. Karimi, T. P. Fitzgerald, N. S. Fisher, A Quantitative Synthesis of Mercury in Commercial Seafood and Implications for Exposure in the United States. *Env. Health Perspect.* **120**, 1512 (2012).
4. D. L. Taylor, N. J. Kutil, A. J. Malek, J. S. Collie, Mercury bioaccumulation in cartilaginous fishes from Southern New England coastal waters: Contamination from a trophic ecology and human health perspective. *Mar. Environ. Res.* **99**, 20–33 (2014).
5. D. Pauly, D. Zeller, "Catch Reconstruction: concepts, design and data sources" in (Sea Around Us (www.seaaroundus.org), 2020).
6. E. M. Sunderland, Mercury exposure from domestic and imported estuarine and marine fish in the US seafood market. *Env. Health Perspect.* **115**, 235 (2007).
7. The National Archives, Business Economic Note 24 -Independent Fishmongers. Available at: http://webarchive.nationalarchives.gov.uk/20100512203600/http://www.hmrc.gov.uk/bens/ben24.htm#h.
8. R. Froese, D. Pauly, FishBase. (2015). Available at: http://www.fishbase.org.
9. N. S. Bloom, On the chemical form of mercury in edible fish and marine invertebrate tissue. *Can. J. Fish. Aquat. Sci.* **49**, 1010–1017 (1992).
10. C. Y. Chen, *et al.*, Mercury bioavailability and bioaccumulation in estuarine food webs in the Gulf of Maine. *Env. Sci. Technol.* **43**, 1804–1810 (2009).
11. K. Buckman, *et al.*, Methylmercury Bioaccumulation in an Urban Estuary: Delaware River, USA. *Estuaries and Coasts* **40**, 1358–1370 (2017).
12. C. H. Conaway, S. Squire, R. P. Mason, A. R. Flegal, Mercury speciation in the San Francisco Bay estuary. *Mar. Chem.* **80**, 199–225 (2003).
13. M. Leermakers, S. Galletti, S. De Galan, N. Brion, W. Baeyens, Mercury in the Southern North Sea and Scheldt estuary. *Mar. Chem.* **75**, 229–248 (2001).
14. M. Horvat, *et al.*, Speciation of mercury in surface and deep-sea waters in the Mediterranean Sea. *Atmos. Environ.* **37**, 93–108 (2003).
15. K. Wang, *et al.*, Subsurface seawater methylmercury maximum explains biotic mercury concentrations in the Canadian Arctic. *Sci. Reports 2018 81* **8**, 1–5 (2018).
16. C. Liu, L. Chen, S. Liang, Y. Li, Distribution of total mercury and methylmercury and their controlling factors in the East China Sea. *Env. Pollut.* **258**, 113667 (2020).
17. J. Yang, H. Kim, C. K. Kang, K. R. Kim, S. Han, Distributions and fluxes of methylmercury in the East/Japan Sea. *Deep Sea Res. Part I Oceanogr. Res. Pap.* **130**, 47–54 (2017).
18. A. T. Schartup, *et al.*, Freshwater discharges drive high levels of methylmercury in Arctic marine biota. *Proc. Natl. Acad. Sci.* **112**, 11789–11794 (2015).
19. E. Chassot, *et al.*, "Length-weight Relationships for Tropical Tunas Caught wit h Purse Seine in the Indian Ocean: Update and Lessons Learned, 2016" (IOTC-2016-WPDCS12-INF05).
20. L. J. Muñoz Abril, "Ecología trófica, diversidad genética y contaminación por mercurio del atún aleta amarilla (Thunnus albacares) en la Reserva Marina de Galápagos y el continente ecuatoriano," Quito: USFQ, 2016. (2016).
21. S. C. Walpole, *et al.*, The weight of nations: an estimation of adult human biomass. *BMC Public Health* **12**, 439 (2012).

22. U.S. EPA, “Chapter 8—Body Weight Studies” in *Exposure Factors Handbook*, National Center for Environmental Assessment, Ed. (2011).
